# Cleavage of tropomodulin-3 by asparagine endopeptidase promotes cancer malignancy by actin remodeling and SND1/RhoA signaling

**DOI:** 10.1186/s13046-022-02411-4

**Published:** 2022-06-28

**Authors:** Binghong Chen, Mengying Wang, Junjun Qiu, Keman Liao, Wenrui Zhang, Qi Lv, Chunhui Ma, Zhongrun Qian, Zhonggang Shi, Rong Liang, Yan Lin, Jiazhou Ye, Yongming Qiu, Yingying Lin

**Affiliations:** 1 Brain Injury Center, Department of Neurosurgery, Ren Ji Hospital, Shanghai Jiao Tong University School of Medicine, Shanghai, 200127 People’s Republic of China; 2 Department of Gynaecology, Obstetrics and Gynaecology Hospital, Fudan University, Shanghai, People’s Republic of China; 3 Department of Radiology, School of Medicine, Tongji Hospital, Tongji University, Shanghai, People’s Republic of China; 4 Department of Orthopedics, Shanghai General Hospital of Shanghai Jiao Tong University, Shanghai, People’s Republic of China; 5 Department of Neurosurgery, Division of Life Science and Medicine, The First Affiliated Hospital of University of Science and Technology of China, Hefei, Anhui People’s Republic of China; 6 Department of Medical Oncology, Guangxi Medical University Cancer Hospital, Nanning, Guangxi People’s Republic of China; 7 Department of Hepatobiliary Surgery, Guangxi Medical University Cancer Hospital, Nanning, Guangxi People’s Republic of China

**Keywords:** Malignancy, Asparagine endopeptidase, Tropomodulin-3, Actin remodeling, SND1

## Abstract

**Background:**

Abnormal proliferation and migration of cells are hallmarks of cancer initiation and malignancy. Asparagine endopeptidase (AEP) has specific substrate cleavage ability and plays a pro-cancer role in a variety of cancers. However, the underlying mechanism of AEP in cancer proliferation and migration still remains unclear.

**Methods:**

Co-immunoprecipitation and following mass spectrometry were used to identify the substrate of AEP. Western blotting was applied to measure the expression of proteins. Single cell/nuclear-sequences were done to detect the heterogeneous expression of Tmod3 in tumor tissues. CCK-8 assay, flow cytometry assays, colony formation assay, Transwell assay and scratch wound-healing assay were performed as cellular functional experiments. Mouse intracranial xenograft tumors were studied in in vivo experiments.

**Results:**

Here we showed that AEP cleaved a ubiquitous cytoskeleton regulatory protein, tropomodulin-3 (Tmod3) at asparagine 157 (N157) and produced two functional truncations (tTmod3-N and tTmod3-C). Truncated Tmod3 was detected in diverse tumors and was found to be associated with poor prognosis of high-grade glioma. Functional studies showed that tTmod3-N and tTmod3-C enhanced cancer cell migration and proliferation, respectively. Animal models further revealed the tumor-promoting effects of AEP truncated Tmod3 in vivo. Mechanistically, tTmod3-N was enriched in the cell cortex and competitively inhibited the pointed-end capping effect of wild-type Tmod3 on filamentous actin (F-actin), leading to actin remodeling. tTmod3-C translocated to the nucleus, where it interacted with Staphylococcal Nuclease And Tudor Domain Containing 1 (SND1), facilitating the transcription of Ras Homolog Family Member A/Cyclin Dependent Kinases (RhoA/CDKs).

**Conclusion:**

The newly identified AEP-Tmod3 protease signaling axis is a novel “dual-regulation” mechanism of tumor cell proliferation and migration. Our work provides new clues to the underlying mechanisms of cancer proliferation and invasive progression and evidence for targeting AEP or Tmod3 for therapy.

**Supplementary Information:**

The online version contains supplementary material available at 10.1186/s13046-022-02411-4.

## Introduction

Tumors constitute a major public health problem worldwide and are the leading causes of death and disability [1]. The high proliferative activity and aggressiveness of malignant tumors are the major causes of rapid tumor development and metastasis leading to poor prognosis [2]. In the case of glioblastoma multiforme (GBM), the most aggressive and pernicious type of pathological intracranial tumor, the extremely proliferative and aggressive nature of the tumor leads to an extremely low survival period, with a 5-year survival rate of only 5.8% postdiagnosis [3–5]. Among its complex mechanisms, cytoskeletal remodeling is an important feature of this malignancy and is involved in cell invasion, adhesion, intra- and extracellular signaling, and the epithelial-mesenchymal transition [6–10]. Interestingly, the process of cytoskeletal remodeling is often accompanied by cell cycle changes that accelerate the cell proliferation [11, 12]. Although the cytoskeleton has a regulatory role in core properties of both tumor invasion and proliferation, the underlying mechanisms are far from clear.

The tropomodulin (Tmod) family is a group of actin-binding proteins which cap actin filaments (F-actin) at the slow growing (pointed) end to prevent actin monomer (G-actin) association or disassociation [13]. This family contains four members (Tmod1-4), each with a distinct expression pattern [14]. Distinctively, Tmod3 is ubiquitously expressed in almost all mammalian cells, hinting at the importance of its global biophysiological functions [15]. Structurally, Tmod3 contains one actin-binding region flanked by two tropomyosin (TM)-binding helix regions (TMBS) on the nitrogen (N)-terminus. At the carbon (C)-terminus, a TM-independent actin-binding site (ABS) is composed of five leucine-repeat regions (LRR) following a nonhomologous α helix [16–18]. The expression of Tmod3 in a wide range of tissues and its unique role compared to that played by other Tmods contribute to its various functions in life activities: maintenance of cytoskeletal stress fibers [19], negative regulation of pseudopodia [20], maintenance of epithelial cell polarity [21, 22], cell secretion [23], platelet development [24] and maturation of oocytes [25]. Studies have reported that Tmod3 is a potential tumor suppressor protein, which is compatible with its structure and functions [26]. However, recent studies have shown that high expression of Tmod3 is significantly positively correlated with the occurrence and development of liver cancer, pancreatic ductal adenocarcinoma and non-small cell lung carcinoma [27–29]. The diverse biological functions of Tmod3 and the inconsistent reports of its effect in tumors have attracted interest in studying the regulatory mechanism of Tmod3 in tumors.

Asparagine endopeptidase (AEP), also known as legumain (LGMN), is a lysosomal cysteine proteinase belonging to the C13 family peptidases [30]. AEP is highly expressed and correlates with poor prognosis and advanced clinical stage in various solid tumors including rectal cancer [31], breast cancer [32, 33], epithelial ovary cancer [34] and gastric cancer [35]. Our previous work showed that, after being cleaved at N311, wild-type p53 can be inactivated by AEP, leading to the loss of the p53 tumor suppressor function in GBM [36]. As a specific asparagine endopeptidase, AEP plays specific regulatory roles by cleaving target proteins, as previously confirmed, especially in neurodegenerative diseases, immunoregulation and tumors [37–42]. However, only a few substrates of AEP in tumors have been discovered so far. Novel substrates and the accompanying pathological functions generated after substrate cleavage need to be clarified.

In the present study, we demonstrate that AEP specifically cleaves Tmod3 at N157 and produces two truncated Tmod3 proteins, which regulate the invasion and proliferation of cancer cells through cytoplasmic actin remodeling and nuclear SND1/RhoA signaling, respectively. The specific cleavage of Tmod3 can be detected in array of solid tumors, and the cleavage of Tmod3 is significantly associated with poor prognosis in high grade glioma (HGG). Thus, our study illustrates a novel mechanism of AEP-mediated tumor progression and suggests the possibility that AEP-Tmod3 might be a novel therapeutic target in cancer. In addition, starting with the goal of understanding unique properties of GBM, we delineate a pan-cancer rule based on specific enzymatic activation of Tmod3 by AEP.

## Materials and methods

### Reagents and chemicals

Dulbecco’s modified Eagle’s medium (DMEM) was purchased from Gibco (Grand Island, NY, USA). The antibodies and assay kits used in the study were listed in the Key Resources Table in the Supplementary file: Supplementary materials and methods. HRP conjugated donkey anti-mouse/rabbit/goat antibodies were purchased from Abcam (Cambridge, UK). All of the other chemicals were purchased from Sigma-Aldrich China (Shanghai, China) or Sangon Biotech (Shanghai, China) unless otherwise described.

### Cell lines and cell culture

The human glioma cell lines (A172 and U87-MG), human embryonic kidney cells (HEK293) were purchased from American Type Culture Collection (ATCC). The human glioma cell lines (U251-MG) and human cervical carcinoma cell line (HeLa) were purchased from the National Collection of Authenticated Cell Cultures. All cells were grown in DMEM supplemented with 10% fetal bovine serum (Gibco) and 100 IU/ml penicillin/streptomycin (Gibco). All cells were grown in a humidified incubator with atmosphere 5% CO_2_ at 37 °C.

### Western blotting

Cell lysates were prepared in RIPA buffer (Solarbio, Beijing, China) containing phenylmethylsulfonyl fluoride (PMSF). Lysates were incubated on ice for 30 min and centrifuged at 12,500 g for 10 min at 4 °C. Protein concentrations of the supernatants were determined with BCA protein assay kit (ThermoFisher Scientific, Waltham, MA, USA). Protein samples were denatured using Loading buffer (with DTT). Proteins were then separated on precast 10% PAGE gels (Yeason, Shanghai, China) and transferred to polyvinylidene fluoride (PVDF) membranes (Millipore, Boston, MA, USA). After blocking in blocking buffer (5% skim milk in TBS with 0.5% Tween-20), membranes were incubated with the primary antibody (listed in the Supplementary materials and methods) overnight at 4 °C. Then the membranes were washed three times with TBST buffer, incubated with the HRP-conjugated appropriate secondary antibodies for an hour at room temperature. Chemiluminescent detection was performed using a ChemiDoc Imaging System (Bio-Rad, Hercules, CA, USA) after adding ECL solution (ThermoFisher Scientific) on the membrane.

### Co-immunoprecipitation and mass spectrometry

For the immunoprecipitation assays, whole-cell extracts were prepared, incubated overnight with the appropriate antibodies, and subsequently incubated with Protein A/G beads (sc-2003, Santa Crus Biotechnology). The beads were washed five times with low-salt lysis buffer, and the immunoprecipitates were eluted in 1 $\times$ SDS Loading Buffer and resolved by SDS-PAGE. Proteins were transferred to PVDF membrane and incubated with the appropriate antibodies. After immunoprecipitation, the protein bands were excised in our laboratory using trypsin-based in-gel protein digestions, and the subsequent mass spectrometry (MS) analysis was conducted at Shanghai Applied Protein Technology Co., Ltd and Oebiotech Co., Ltd (Shanghai, China).

### Patient information and tissue specimens

Seventy-two freshly frozen glioma tissues (low grade glioma (LGG): *n* = 24, HGG: *n* = 48) and eight normal brain tissues were obtained from the Department of Neurosurgery, Ren Ji Hospital, School of Medicine, Shanghai Jiao Tong University; Thirty cervical cancer tissues were obtained from the Obstetrics and Gynecology Hospital of Fudan University. Twenty-five hepatocellular carcinomas tissues were obtained from Guangxi Medical University Cancer Hospital. All specimens were obtained with written informed consent from the patients. All of these samples were obtained at the initial diagnosis. The study was approved by the ethics committee of the Ren Ji Hospital, School of Medicine, Shanghai Jiao Tong University (IRB number, RA-2021–212), the ethics committee of Obstetrics and Gynecology Hospital of Fudan University (IRB number, 2021–14) and the ethics committee of Guangxi Medical University Cancer Hospital (IRB number, KY2020025).

### Single-cell isolation and single-cell/nuclear-seq library preparation and data preprocessing

Single-cell/nuclear RNA sequencing was performed on a tumor biopsy specimen collected from patients with GBM or cervical cancer treated at Ren Ji Hospital, Shanghai Jiao Tong University, or Obstetrics and Gynecology Hospital of Fudan University School of Medicine. The biopsy specimens from the patient were banked for research purposes following consent through an IRB-approved protocol. Libraries were prepared following the manufacturer’s protocol of Chromium Next GEM Single Cell 3ʹ Reagent Kits v3.1. The Cell Ranger software pipeline (version 3.1.0) provided by 10 × Genomics was used to demultiplex cellular barcodes, map reads to the genome and transcriptome using the STAR aligner, and down-sample reads as required to generate normalized aggregate data across samples, producing a matrix of gene counts versus cells. We processed the unique molecular identifier (UMI) count matrix using the R package Seurat (version 3.1.1). The sequencing and bioinformatics analysis were performed by Oebiotech Co., Ltd. (Shanghai, China). This is described in detail in the Supplementary file: Supplementary materials and methods.

### Plasmids construction and plasmid DNA transfection

Flag-tagged Tmod3 and its N/C-terminal truncation or corresponding point mutants were cloned into pHY023 vector (Hanyin Biotech, Shanghai, China). ZsGreen1-tagged Tmod3 and its N/C-terminal truncation and NES or NLS mutants were cloned into pHY009 vector (Hanyin Biotech). AEP or C189S-AEP was cloned into pcDNA3.1. Related primers were listed in Supplementary Table 1. All PCR products were confirmed by Sanger sequencing (Genewiz, Suzhou, China). For plasmid DNA transfection, Lipofectamine 2000 (Invitrogen, Carlsbad, CA, USA) was used. Transfection complexes (5 μl transfection reagent to a total volume of 150 μl Opti-MEM were mixed with 2.5 μg recombinant plasmid DNA to a volume of 150 μl) were left at room temperature for 15 min to combine fully. The mixture was then added to the prepared adherent cells with a total volume of 1 ml complete medium. Medium change was performed after 24 h of incubation in a cell culture incubator. The effects of gene knockdown or overexpression were confirmed by fluorescence or western blotting.

### Construction of cell lines with specific gene knockdown or overexpression

Human *TMOD3*/*LGMN*-specific short hairpin RNA (shRNA) and scramble shRNA (shCtrl), Tmod3 truncations (*TMOD3*-1–157 and *TMOD3*-158–352), human *SND1* overexpression and negative controls (pHY009-ZsGreen1 and pHY023) were purchased from Hanyin Biotech (Shanghai, China). Recombinant plasmids were packaged by lentivirus to infect the corresponding tumor cell lines. Briefly, on day 1 cells were plated in a low density (50, 000 cells/well) in a 6-well plate. This was done in replicates of three per cell line in addition to one negative transfection control per cell line. On day 2 lentiviral particles were added together with polybrene (5 μg/ml) in fresh media (1:1). Media change to complete media without polybrene after 6 h. Puromycin (1 μg/ml) selection for successfully transduced cells started on day 5. The shRNA targeting *TMOD3* are listed in the Supplementary Table 1.

### siRNA transfection

For siRNA-mediated knockdown, cells were transfected with 10 nM of *SND1*-specific siRNAs or a scrambled siRNA sequence (siCtrl) as a control (purchased from Ribobio (Guangzhou, China), see Supplementary Table 1) using Lipofectamine 2000 on the day of plating and subjected to subsequent analysis.

### Prokaryotic expression and proteins purification

Related proteins with 6 $\times$ His tags were expressed in the prokaryotic expression system with pET28a as the expressing vector, BL21 (DE3) as the competent cells, and isopropyl-β-d-thiogalactoside (IPTG, 0.1 mM) as the inducer. Expressed proteins were confirmed by Coomassie brilliant blue staining and purified with Nickel Magnetic Beads (LSKMAGH02, Millipore) according to the manufacturer’s manual.

### Determination of the F-/G-Actin ratio

The F-/G-actin ratio was examined using immunofluorescence (IF) and western blotting. For IF, F-actin was labeled with YF633 Dye Phalloidin Conjugates (200 T/ml, Cat. YP0053, US EVERBRIGHT INC, Suzhou, China). G-actin was labeled with Deoxyribonuclease I, Alexa FluorTM 594 Conjugate (0.3 μM, D12372, Invitrogen) for 20 min at room temperature in dark. For western blotting, immunoprecipitation assay lysis buffer (Cat. P0013K, Beyotime Biotechnology, Shanghai, China) was employed to lyse cells on ice for 10 min, followed by centrifugation at 15,000 g for 30 min. Soluble actin (G-actin) was collected in the supernatant. The insoluble F-actin in the pellet was resuspended in lysis buffer with an equal volume of buffer 2 (1.5 mM guanidine hydrochloride, 1 mM sodium acetate, 1 mM CaCl_2_, 1 mM adenosine triphosphate, and 20 mM Tris HCl, pH 7.5) and incubated on ice for 1 h, with gentle mixing every 15 min, to convert F-actin into soluble G-actin. The samples were then centrifuged at 15,000 g for 30 min, F-actin was collected in the supernatant. Samples from the supernatant (G-actin) and pellet (F-actin) fractions were analyzed by western blotting using an antibody specific for actin.

### Fluorescence assays for actin polymerization

Elongation rates at actin filament pointed-end were measured using the Actin Polymerization Biochem Kit (Cat. BK003, Cytoskeleton, Inc, Denver, CO, USA) according to the manual. Briefly, dilute rabbit muscle actin to 2 uM (10% Pyrene labeled) with A-buffer (General Actin Buffer (5 mM Tris–HCl pH 8.0, 0.2 mM CaCl_2_) supplemented with 0.2 mM ATP and 1 mM DTT). Leave on ice for 1 h to depolymerize actin oligomers. Then centrifuge at 14,000 rpm at 4 ℃ for 0.5 h to remove residual nucleating centers. Pipet 90 μl of the actin dilution into two wells of a black assay 96-well plate. Pipet 90 μl of General Actin Buffer into two wells as control samples. Place 96-well plates into the fluorescent spectrophotometer (with an excitation wavelength of 365 nm and emission wavelength of 407 nm) and read the samples for 1 h to establish a baseline fluorescent measurement. After that, add 10 μl of 10 × Actin Polymerization Buffer (500 mM KCl, 20 mM MgCl_2_, and 10 mM ATP in 100 mM Tris, pH 7.5) supplemented with purified proteins to each well and gently mix. At last, return the plate to the spectrophotometer and read the fluorescence every 30 s for 1 h. A time polymerization fluorescent enhancement curve is drawn.

### Nuclear and cytoplasmic protein extraction

A Nuclear and Cytoplasmic Protein Extraction Kit (Cat. P0027, Beyotime Biotechnology, Shanghai, China) was used to extract nuclear and cytoplasmic proteins. Briefly, prepared cells were added with moderate cytoplasmic protein extraction reagent A (200 μl / 20 μl cells) supplemented with 1 mM PMSF. Vortex and lysis for 10–15 min on the ice. Then add 10 μl of cytoplasmic protein extraction reagent B and strong vortex for 5 s, ice for 1 min. Centrifuge at 14, 000 g at 4 ℃ for 5 min, pipet the supernatant to a clean EP tube as the cytoplasmic protein. For precipitation, completely aspirate the remaining supernatant, and add 50 μl of nucleoprotein extraction reagent supplemented with PMSF. Vortex and lysis for 30 min on the ice. Centrifuge at 14, 000 g at 4 ℃ for 10 min, pipet the supernatant to a clean EP tube as the nuclear protein.

### Cell immunofluorescence (IF)

IF experiments were performed. Briefly, 2 × 10^5^ cells were seeded on coverslips in each well of a 6-well plate. The cells were washed 3 times with phosphate buffer saline (PBS) before fixation with 4% paraformaldehyde. The cells were blocked with PBS containing 0.3% TritonX-100 and 5% bovine serum albumin (BSA) for 30 min. Antibodies were incubated at 4 °C overnight. The cells were washed 6 times with PBST (PBS containing 0.3% TritonX-100) for a total of an hour and incubated with secondary antibodies for 1 h at room temperature. The samples were observed with a Zeiss laser scanning confocal microscope (ZEISS LSM 510 Meta). Single sections are shown. Images were processed (colored and merged) with Zeiss (LSM 510) software.

### Cell counting kit-8 (CCK-8) assay, flow cytometry for apoptosis detection, colony formation assay, transwell assay, and scratch wound-healing assay

The cell functional experiments were conducted as previously described [36], and described in detail in the Supplementary file: Supplementary materials and methods.

### In vivo experiments

Male-nude mice (4 to 5 weeks old) purchased from Lingchang Biotech (Shanghai, China) were maintained and treated in accordance with the guidelines approved by the Institutional Animal Care and Use Committee of School of Medicine, Shanghai Jiao Tong University (IRB number, B-2019–003). Prepared U87-MG cells (1 $\times$ 10^6^ / 5 μl) were injected into the intracerebral region of mice. When mice showed significant weight loss or symptoms of neurological impairment, magnetic resonance imaging (MRI) was applied to detect intracranial tumors. Optional 3 mice were sacrificed on the day of MRI detection and brain tissues were taken for further experiments (described in detail in the Supplementary file: Supplementary materials and methods). The remaining mice were kept until they died on their own to calculate the overall survival.

### Statistical analysis

All immunoblotting assays had been performed at least thrice to ensure the repeatability of the experiments. All statistical analysis was generated based on at least three independent experiments. Data for all experiments were analyzed with GraphPad Prism 7 or SPSS 14.0. Two-sided *P* values of less than 0.05 were considered statistically significant.

## Results

### AEP binds to Tmod3 and specifically cleaves Tmod3 at N157.

**Fig. 1:**
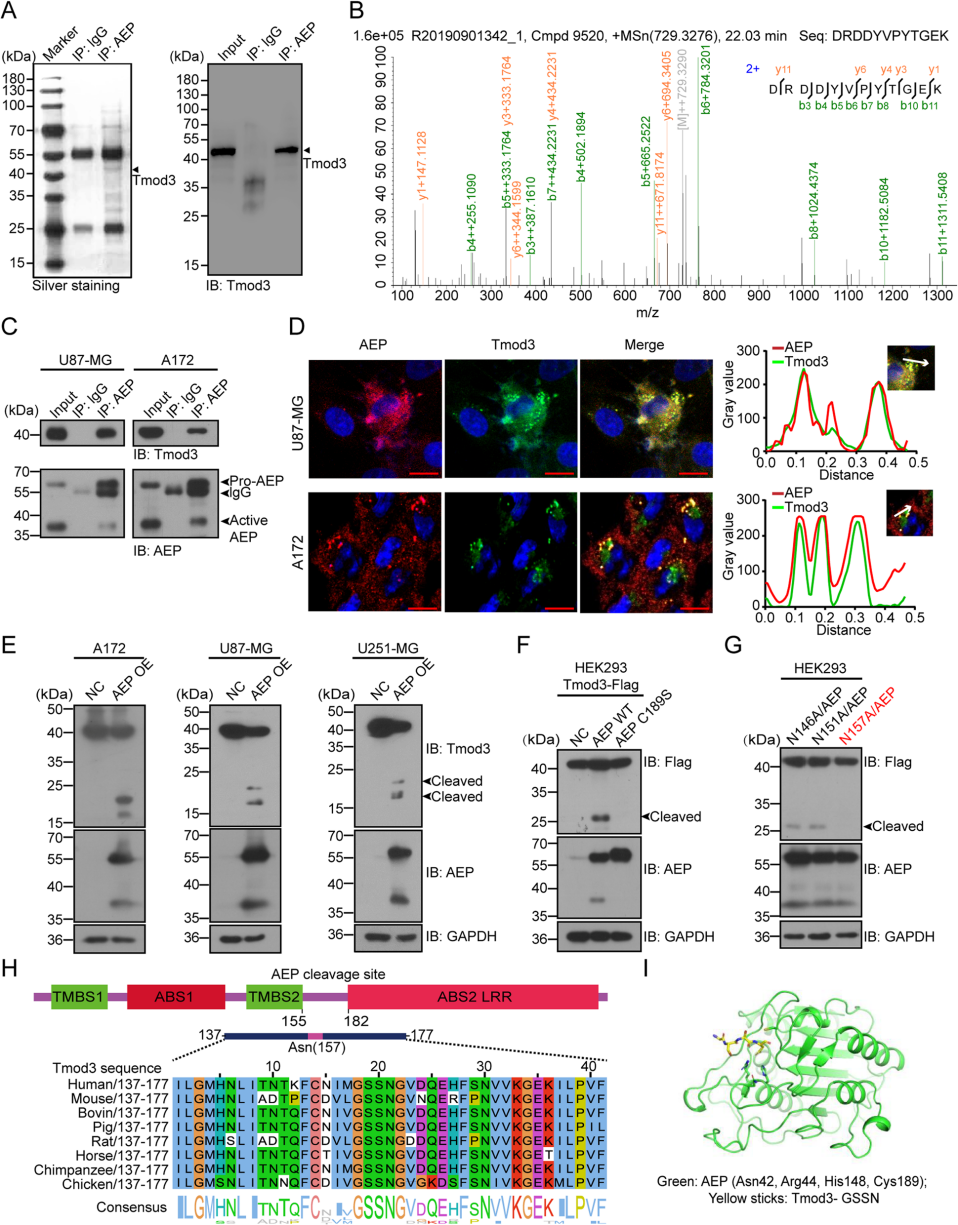
AEP binds to Tmod3 and specifically cleaves Tmod3 at N157.** A** Left panel: silver-stained gel showing immunoprecipitated AEP or lgG and their bound proteins from mouse embryonic fibroblasts (MEFs); right panel: immunoblot showing Tmod3 exists in AEP immunoprecipitants. Arrowhead indicates Tmod3. **B** Mass spectrometry analysis of proteins interacting with AEP. The detected MS/MS peptide spectra of Tmod3 are shown. **C** Endogenous co-IP and western blotting of the AEP/Tmod3 interaction in U87-MG and A172 cells. **D** Representative confocal images showing the colocalization of AEP (red) and Tmod3 (green) in U87-MG and A172 cells by immunofluorescent staining. Scale bar, 20 μm. Co-localization tracer profile along the line (white arrows) is indicated as merged image (right panel). **E** Immunoblots of Tmod3, AEP and GAPDH in A172, U87-MG and U251-MG cells with or without AEP OE. NC = negative control, cells expressing empty vector, OE = overexpression. **F** Immunoblots of Flag-tagged Tmod3, AEP and GAPDH in HEK293 cells co-transfected with Flag-Tmod3 and wild-type AEP or the AEP C189S mutant. NC, negative control, cells transfected with empty vector. **G** Immunoblots of Flag-tagged Tmod3, AEP and GAPDH in HEK293 cells co-transfected with AEP and panels of Tmod3 point mutants. **H** Tmod3 amino acid sequence alignment among different species. **I** Emulated domains of Tmod3-GSSN- interacting with AEP enzymatic center. Green (AEP enzymatic center), yellow sticks (Tmod3-GSSN-)

To screen substrates of AEP, we conducted proteomic analysis with immunoprecipitated AEP complex. Silver staining and mass spectrometry were performed, and Tmod3 was thus identified as a candidate substrate (Fig. 1A-B, Supplementary Table. 2). We found that plenty of cytoskeleton related proteins identified in our data, such as Vimentin, Cortactin, and IQ Motif Containing GTPase Activating Protein 1 (IQGAP1), also have been revealed in gastric cancer [43]. More, endogenic coimmunoprecipitation (co-IP) assays revealed that AEP interacted with Tmod3 in GBM cells (Fig. 1C). Immunofluorescent (IF) staining indicated that Tmod3 colocalized with AEP around the nucleus (Fig. 1D). AEP has been reported to act as an important lysosomal protease that regulates protein substrate homeostasis [44]. Detection of lysosomal status with a lysosomal probe in AEP-knockdown GBM cells showed that AEP knockdown significantly activated lysosomes (Fig. S1A). To further investigate whether the interaction of AEP with Tmod3 occurs in the lysosomes or not, triple immunofluorescence was used to detect co-localization of AEP/Tmod3 with the lysosomal biomarker LAMP2. The result showed that AEP was highly co-localized with Tmod3, but both were only marginally co-localized with LAMP2 (Fig. S1B), suggesting that the interaction of AEP with Tmod3 mainly occurs outside lysosomes, which is consistent with our previous finding [36].

Next, we detected the cleavage of Tmod3 in GBM cells with or without high AEP expression. Two fragments with molecular weight between 15 and 25 kDa were found in cells with high AEP expression (Fig. 1E). To further explore whether the cleavage of Tmod3 was dependent on AEP enzymatic activity, wild-type (WT)-AEP or enzymatically inactive AEP (C189S mutant) was transiently co-expressed with carbon terminal Flag-tagged Tmod3 (Tmod3-Flag) in HEK293 cells. Only cells co-transfected with WT-AEP could be detected with cleaved Tmod3, while the cells transfected with AEP-C189S failed to exhibit the cleavage of Tmod3. These results suggested that AEP was responsible for eliciting Tmod3 proteolytic cleavage (Fig. 1F).

AEP is known to be strictly selective in cleaving asparagine [30]. To identify the precise cleavage site in Tmod3, we generated a panel of carbon (C)-terminal Flag-tagged Tmod3 mutants with asparagine (N) to alanine (A) substitution mutations. The results indicated that only the cells co-transfected with the Tmod3-N157A mutant and AEP failed to produce the cleavage product, definitively indicating that N157 was the specific cleavage site at which AEP cleaved Tmod3 (Fig. 1G and Fig. S2A). Through multispecies sequence alignment, N157 was found to be species conserved and precisely fit into the AEP enzymatic center (Fig. 1H-I).

As AEP cleaves Tmod3 at site N157 between the TMBS2 and ABS2 functional domains of Tmod3, Flag-tagged C-terminal truncated Tmod3 (amino acids, 1–157, referred to as tTmod3-N) and N-terminal truncated Tmod3 (amino acids, 158–352; referred to as tTmod3-C) were constructed (Fig. S2B). The interactions between AEP and two truncations were detected by co-IP, and it was found that tTmod3-C played a critical role in interacting with AEP (Fig. S2B).

Considering that specific mutations in a protein may affect its biological function, we searched Cancer Cell Line Encyclopedia (https://portals.broadinstitute.org) and collected sixteen natural missense mutations of Tmod3 in cancer. The corresponding mutant Tmod3 was co-transfected with AEP into HEK293 cells. Most of them and wild-type Tmod3 differed negligibly, except for the Tmod3 T256S, V262M and L303M mutant, which significantly promoted the cleavage of Tmod3 (Fig. S2C-D). In addition, Tmod3 was reported to be phosphorylated at Ser71 upon insulin-stimulated Akt2 activation, with its function significantly facilitated in actin remodeling [23]. However, phosphorylation of Ser71 did not affect the cleavage of Tmod3 (Fig. S2E). And that, we also examined the AEP-induced cleavage of other Tmod family proteins and found similar cleavage of Tmod1 or Tmod2, but not of Tmod4 (Fig. S2F). Taken together, these results indicate that AEP interacts with Tmod3 and cleaves at N157, generating two Tmod3 truncations with complete functional domains in cancer cells.

### Tmod3 is highly expressed in many solid cancers and is associated with poor patient prognosis.

To investigate the expression of Tmod3 in diverse tumors and its correlation with prognosis. We searched the Gene Expression Profiling Interactive Analysis (GEPIA). Tmod3 was found to be upregulated in diverse types of tumors such as cholangio carcinoma (CHOL), pancreatic adenocarcinoma (PAAD) and stomach adenocarcinoma (STAD), compared to the corresponding normal tissues (Fig. S3A). We also searched the Gene Expression Omnibus (GEO) database, and analysis of the GSE 4290 dataset confirmed that Tmod3 was upregulated in HGG compared with LGG and normal brain tissues (Fig. S3B). High Tmod3 expression is associated with poor patient prognosis in many types of tumors, including GBM and LGG, cervical squamous cell carcinoma and endocervical adenocarcinoma (CESC), lung adenocarcinoma and adenosquamous carcinama (LUAD and LUSC), PAAD and bladder urothelial carcinoma (BLCA) (Fig. S3C-G).

**Fig. 2:**
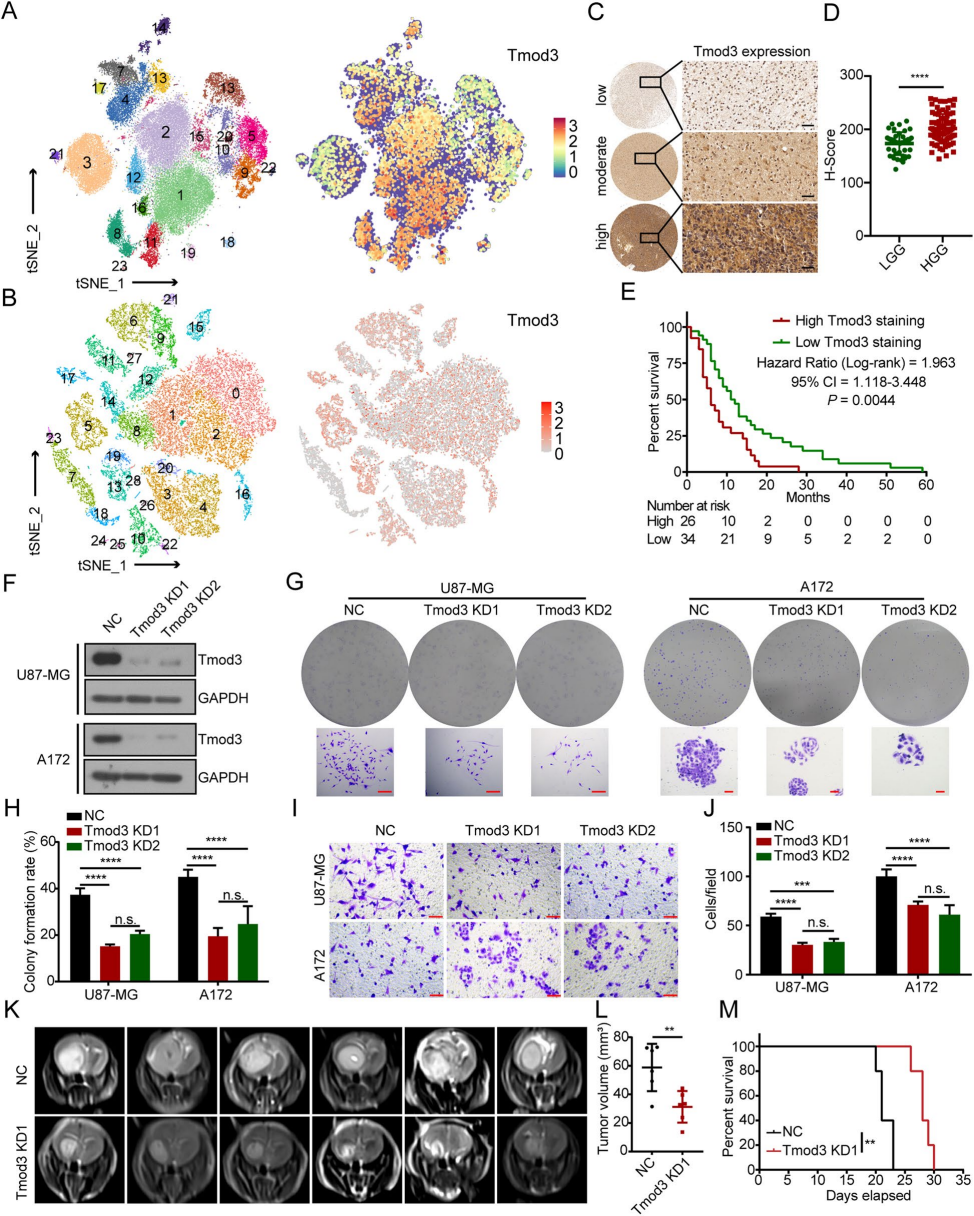
Tmod3 is highly expressed in many solid tumors and is associated with poor patient prognosis.** A, B** snRNA-seq with GBM cells (**A**) and scRNA-seq with cervical cancer cells (**B**) populations colored by clusters, number of cells analyzed in each cluster in the legend and the expression of Tmod3 in each cluster. The darker the red color, the higher the expression of the corresponding gene in that cell. **C** Representative images of low, moderate and high IHC staining of Tmod3 in glioma tissue array. Scale bar, 40 μm. **D** Histochemistry score (H-score) of Tmod3 in LGG and HGG of the tissue array; the data are shown as the mean ± s.d. **E** Kaplan–Meier survival plots showing survival rates of the patients in the indicated groups with high or low Tmod3 H-scores. Comparisons of survival curves were performed with Log-rank test. **F** Immunoblots of Tmod3 and GAPDH in U87-MG and A172 cells with or without Tmod3 KD. **G, H** Colony formation assay of U87-MG and A172 cells with or without Tmod3 KD. Scale bar, 100 μm. Statistical analysis of colony formation rate was done (H). Triplicates in each group are the bases for the presented mean ± s.d. **I, J** Transwell assay of U87-MG and A172 cells with or without Tmod3 KD. Scale bar, 100 μm. Triplicates in each group, data are presented as mean ± s.d. **K** Representative coronal MRI of xenograft GBM tumors orthotopically inoculated with U87-MG cells with or without Tmod3 KD on the 20^th^ day postoperation (n = 8 per group). **L** The tumor volume was calculated in each group using the Coniglobus formula method. The data are presented as the mean ± s.d. **M** Kaplan–Meier plot analysis of the OS of mice in the indicated groups (n = 5 per group). Comparisons of survival curves were performed with Log-rank test. NC = negative control, cells expressing scramble shRNA. KD = knockdown, cells expressing Tmod3 shRNA. ^**^P < 0.01, ^***^P < 0.001, ^****^P < 0.0001, n.s. = no significance

Since malignant tumors often exhibit marked heterogeneity, in order to investigate the expression of Tmod3 in different cell subtypes within the tumor, we measured the expression of Tmod3 by performing single-nuclear RNA-seq (snRNA-seq) with 6 cases of GBM and single-cell RNA-seq (scRNA-seq) with 8 cases of cervical cancer tissues. After single-nucleus/cell suspension preparation and single cDNA library sequencing, the t-distributed stochastic neighbor embedding (t-SNE) algorithm distinguished 23 clusters and 28 clusters of cells in GBM and cervical cancer, respectively. The results indicated that Tmod3 was highly and ubiquitously expressed in most clusters (Fig. 2A-B). We further detected the expression of Tmod3 by immunohistochemical staining in a tissue array of 123 human glioma specimens. Tmod3 was found to be highly upregulated in HGG compared with LGG (Fig. 2C-D). Patients whose Tmod3-stained samples had a high H-score presented with poorer overall survival (OS) (60 of 123 patients with definitive survival follow-up data, *P* = 0.0044, HR = 1.963, 95% CI, 1.118–3.448. Figure 2E, Supplementary Table 3). Western blotting analysis of glioma tissues consistently showed that Tmod3 was significantly upregulated in HGG compared to LGG and normal brain tissues (Fig. S3H).

To identify the functions of Tmod3 in cancer cells, we constructed Tmod3-knockdown (KD) U87-MG and A172 cells using lentivirus. The effects of Tmod3 KD were confirmed by western blotting (Fig. 2F). Lysosomal probe detection indicated that Tmod3 KD had a limited effect on lysosomes (Fig. S4A). The CCK-8 assay indicated that Tmod3 KD significantly inhibited the proliferation of GBM cells (Fig. S4B). Flow cytometry results suggested that Tmod3 KD led to increased apoptosis rates in both cells (Fig. S4C-D). The colony formation ability was significantly impaired in the cells with Tmod3 KD (Fig. 2G-H). In addition, the invasive ability of U87-MG and A172 cells was also inhibited by Tmod3 KD, as detected by Transwell assay and scratch wound-healing assay (Fig. 2I-J and Fig. S4E-F). Simultaneously, in vivo animal models suggested that Tmod3 KD reduced the tumorigenesis of GBM (Fig. 2K-L and Fig. S4G). Tmod3 KD reduced weight loss and prolonged the OS of tumor-bearing mice (Fig. S4H and Fig. 2M). Immumohistochemical staining (IHC) indicated that biomarkers of proliferation (Ki-67) and invasion (Vinculin) were significantly reduced by Tmod3 KD (Fig. S4I-J). Taken together, the results suggested that Tmod3 may exert deleterious effects in different types of solid tumors, and that suppression of Tmod3 significantly inhibits the malignancy-related functions of GBM cancer cells in vitro and in vivo.

### AEP-produced tTmod3 is found in many types of solid tumors and is highly associated with poor prognosis in patients with GBM.

**Fig. 3:**
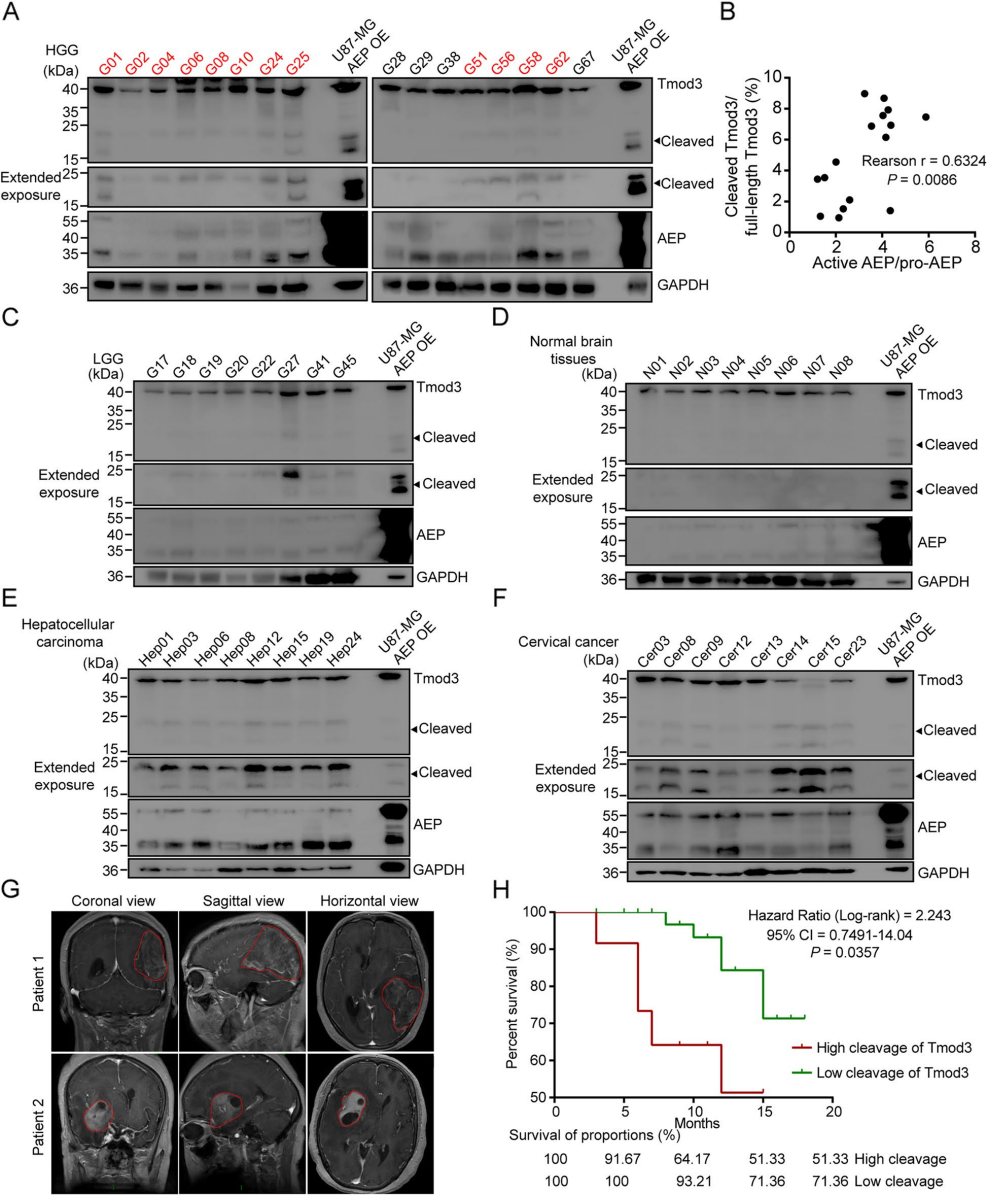
AEP-produced tTmod3 exists in many types of solid tumors and is highly associated with poor prognosis in patients with GBM. **A** Immunoblots of Tmod3, AEP and GAPDH in HGG. Tissues with obvious Tmod3 cleavage bands are marked in red. **B** Correlation analysis of AEP activation and cleavage of Tmod3 in tissues of HGG. Pearson r = 0.6324, *P* = 0.0086. **C** Immunoblots of Tmod3, AEP and GAPDH in LGG. **D** Immunoblots of Tmod3, AEP and GAPDH in normal brain tissues. **E** Immunoblots of Tmod3, AEP and GAPDH in hepatocellular carcinoma tissues. **F** Immunoblots of Tmod3, AEP and GAPDH in cervical cancer tissues. **G** Enhanced brain MRI images of GBM tissues with or without cleavage of Tmod3. The general extent of the tumor is circled in red. Patient 1: with high cleavage of Tmod3; Patient 2: without cleavage of Tmod3. **H** Kaplan–Meier survival plots showing the survival rates of the two groups of patients with or without cleavage of Tmod3. Comparisons of the survival curves were performed with Log-rank tests

In biochemical experiments, Tmod3 was found to be cleaved by AEP in cancer cells. Does this event truly occur in clinical tumor tissues, and if it does, then what does this cleavage indicate about clinical diagnosis and treatment? We collected 72 human freshly frozen glioma tissues (LGG, 24; HGG, 48), 8 normal brain tissues, 25 hepatocellular carcinoma and 30 cervical cancer tissues to detect the cleavage of Tmod3 by western blotting. Obviously, bands indicating specific Tmod3 cleavage were definitively detected in the HGG tissues (25.0%, 12 of 48 cases, Fig. 3A and Fig. S5A-B), and there was a positive correlation between AEP activation and cleavage of Tmod3 (Fig. 3B). However, the cleavage of Tmod3 was nearly undetectable in LGG and normal tissues (Fig. 3C-D and Fig. S5C). The cleavage of Tmod3 was also detected in the hepatocellular carcinoma tissues (32.0%, 8 of 25 cases, Fig. 3E and Fig. S5D) and the cervical cancer tissues (26.7%, 8 of 30 cases, Fig. 3F and Fig. S5E). We analyzed the correlation between the high cleavage of Tmod3 and the clinical characteristics of HGG patients, and the results were shown in Supplementary Table 4. We found that age, sex, cancer recurrence, WHO tumor grade and isocitrate dehydrogenase 1 (IDH1) mutation were without difference in patients with or without Tmod3 cleavage. Tumors with high Tmod3 cleavage tended to be invasive in multiple lobes (Fig. 3G). Moreover, compared to patients with lower cleavage of Tmod3, patients with obvious cleavage of Tmod3 presented with shortened OS (*n* = 48, *P* = 0.0357, HR = 2.243, 95% CI, 0.7491–14.04, Fig. 3H, Supplementary Table 4). Thus, these results strongly indicated that AEP cleavage of Tmod3 is a common event in many types of solid tumors and that the cleavage of Tmod3 is a promising biomarker for indicating poor prognosis in patients with GBM.

### Tmod3 truncations produced by AEP cleavage promote GBM proliferation and invasion.

**Fig. 4:**
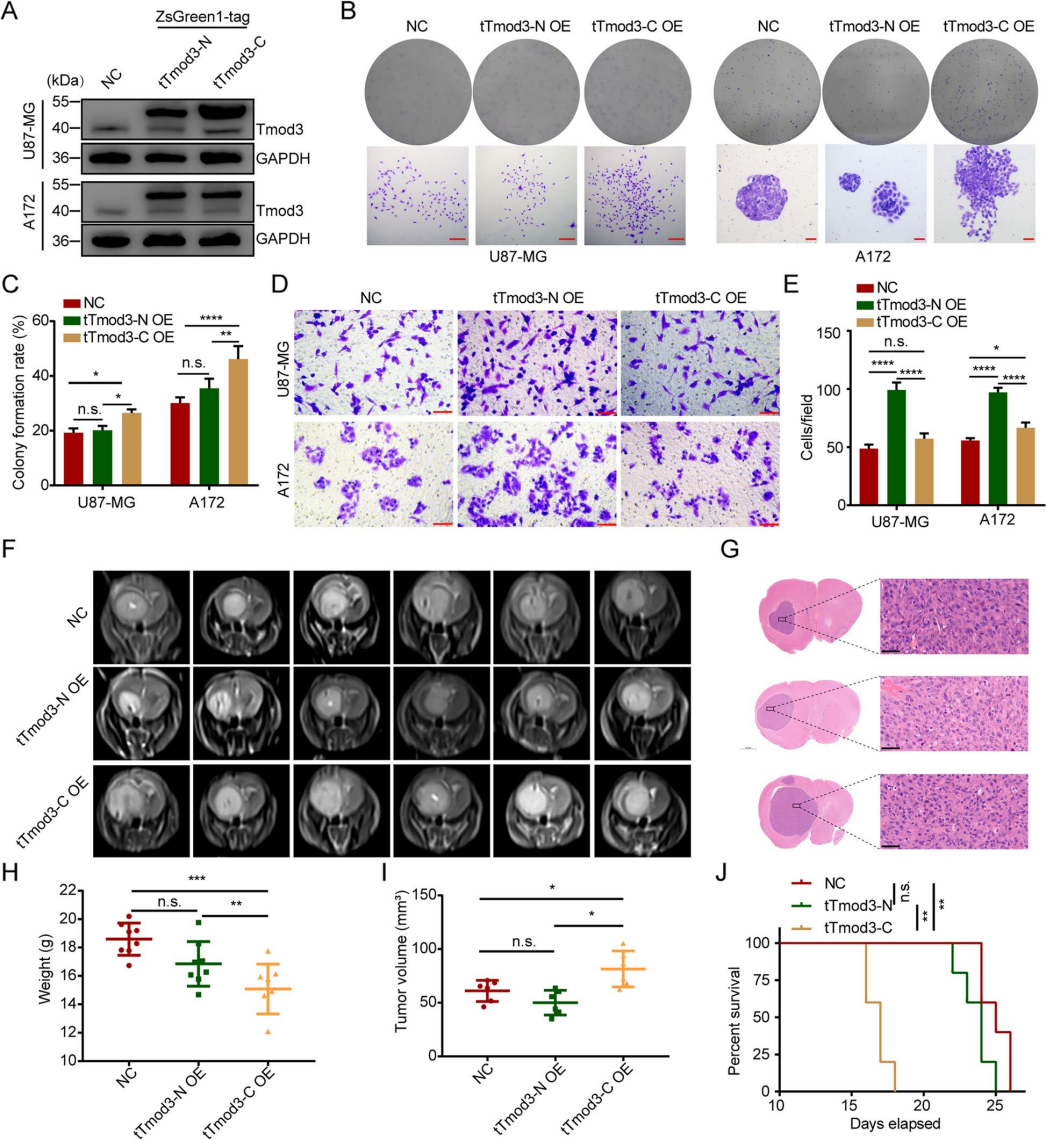
Tmod3 truncations produced by AEP cleavage promote GBM proliferation and invasion. **A** Immunoblots of Tmod3 and GAPDH in U87-MG and A172 cells with or without ZsGreenl-tagged Tmod3 truncations overexpression. **B, C** Colony formation assay of U87-MG and A172 cells with or without ZsGreenl-tagged Tmod3 truncations overexpression. Scale bar, 100 μm. Statistical analysis of the colony formation rate (C). Triplicates in each group are the bases for the presented mean ± s.d. **D, E** Transwell assay of U87-MG and A172 cells with or without ZsGreenl-tagged Tmod3 truncations overexpression. Scale bar, 50 μm. Triplicates in each group are the bases for the presented mean ± s.d. **F** Representative coronal MRI of xenograft GBM tumors orthotopically inoculated with U87-MG cells with or without ZsGreenl-tagged Tmod3 truncations overexpression on the 16^th^ day postoperation (n = 8 per group). **G** Representative H&E images from every group are shown. Scale bar, 40 μm. **H** The weight of the mice in every group on the day of MRI detection. The data are presented as the mean ± s.d. **I** The tumor volume was calculated for every group using the Coniglobus formula. The data are presented as the mean ± s.d. **J** Kaplan–Meier survival plots analysis of the OS for mice of the indicated groups (*n* = 5 per group). Comparison of survival curves was performed with Log-rank tests. NC, negative control, cells expressing ZsGreen1. OE = overexpression, cells expressing ZsGreen1-tTmod3-N or ZsGreen1-tTmod3-C. ^*^P < 0.05, ^**^P < 0.01, ^***^P < 0.001, ^****^P < 0.0001, n.s. = no significance

Since truncations of Tmod3 are present in tumor tissues and are prognostically relevant, what is their function? To answer this question, U87-MG and A172 cells with tTmod3-N or tTmod3-C overexpression (OE) were constructed (Fig. 4A). The promoted proliferative ability of cells with tTmod3-C OE was validated by colony formation assays (Fig. 4B-C) and CCK-8 assays (Fig. S6A). In contrast, in the invasion assays, compared with negative control (NC) cells, cells with tTmod3-N OE were found to have significant elevated motility (Fig. 4D-E). In the in vivo experiments, the group of mice with tTmod3-C OE exhibited significantly enhanced tumor progression (Fig. 4F-I), and the OS of the tumor-burden mice was reduced compared to that of the NC group of mice (Fig. 4J). Next, we measured the expression of Tmod3, Ki-67 and Vinculin in related groups of mice. Consistently, Ki-67 was significantly upregulated in the tTmod3-C OE group, while Vinculin was significantly upregulated in the tTmod3-N OE group (Fig. S6B-C). In addition, in an unexpeted finding, tTmod3-C was found to have markedly increased nuclear accumulation (Fig. S6B). Together, these results suggested that two truncations of Tmod3 may promote GBM cancer cell malignancy in different ways: tTmod3-C mainly promotes tumor proliferation, while tTmod3-N primarily promotes invasion.

### AEP promotes GBM progression by cleaving Tmod3 in vitro and in vivo

**Fig. 5:**
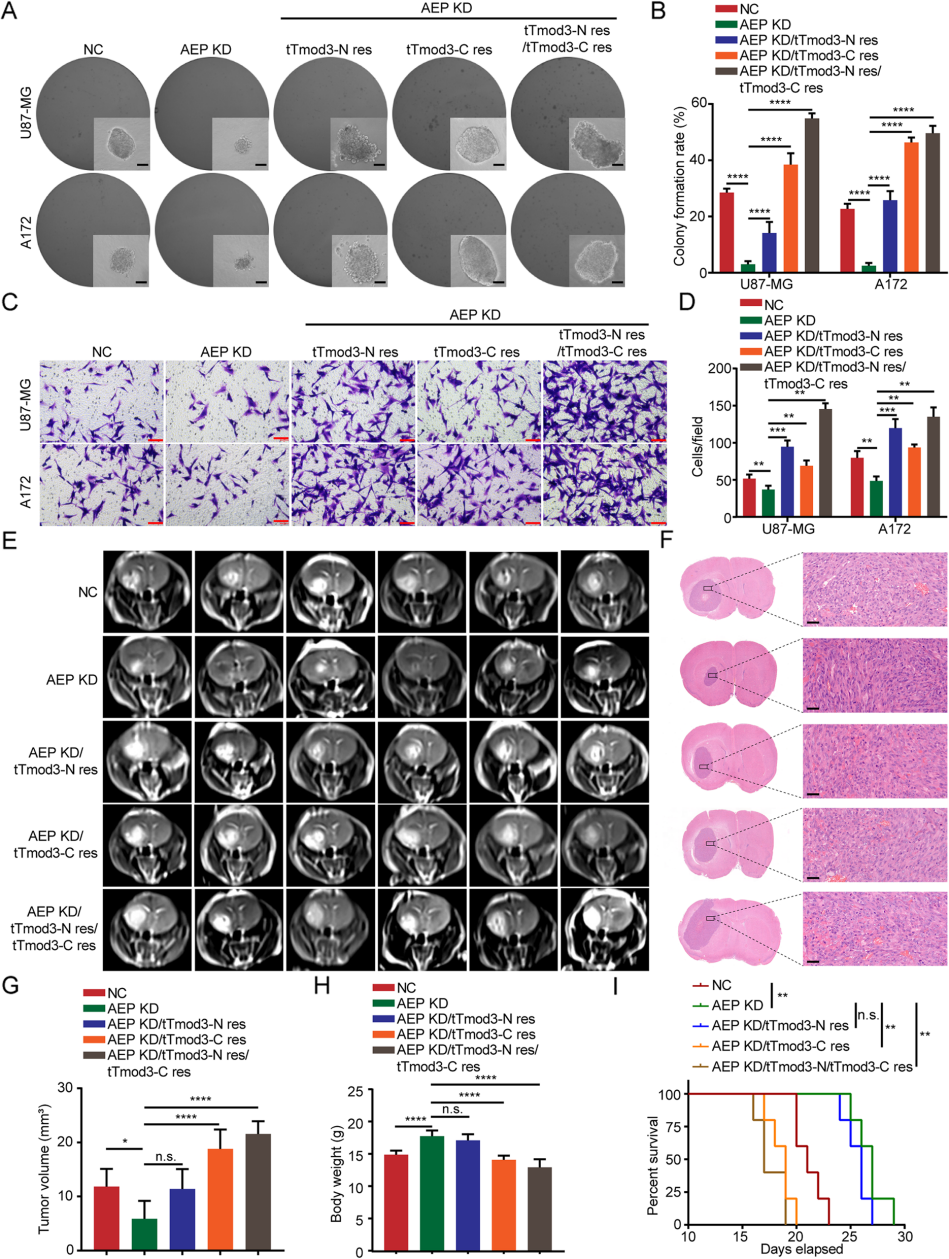
AEP promotes GBM progression by cleaving Tmod3 in vitro and in vivo.** A** Soft agar colony formation assay of U87-MG and A172 cells with NC, AEP KD, AEP KD/tTmod3-N res, AEP KD/tTmod3-C res or AEP KD/tTmod3-N res/tTmod3-C res treatment. Scale bar, 100 μm. Triplicates in each group. **B** Colony formation rate of the indicated group of U87-MG and A172 cells; data are shown as the mean ± s.d. The *P* values of all statistical analyses were determined using a two-sided Student’s t-test, comparing the data of each group with the AEP KD group. **C** Transwell assay of U87-MG and A172 cells with NC, AEP KD, AEP KD/tTmod3-N res, AEP KD/tTmod3-C res, AEP KD/ tTmod3-N res/tTmod3-C res treatment. Scale bar, 100 μm. Triplicates in each group. **D** Cells/field are shown as the mean ± s.d. **E** Representative coronal MRI detection of xenograft GBM tumors orthotopically inoculated with U87-MG cells with NC, AEP KD, AEP KD/tTmod3-N res, AEP KD/tTmod3-C res, AEP KD/tTmod3-N res/tTmod3-C res treatment on 16^th^ day postoperation (*n* = 8 per group). **F** Representative H&E images of every group are shown. Scale bar, 40 μm. **G** The tumor volume was calculated using the Coniglobus formula in every group (*n* = 8 per group). The data are presented as the mean ± s.d. **H** The weight of mice in the indicated groups on the day of MRI detection. **I** Kaplan–Meier survival plots analysis of the OS of mice in the indicated groups (*n* = 5 per group). Comparisons of survival curves were performed with Log-rank tests. NC = negative control, cells expressing scramble shRNA. KD = knockdown, cells expressing AEP shRNA. ^**^P < 0.01, ^****^P < 0.0001, n.s. = no significance

To investigate whether the elevated proliferation and motility mediated by AEP is dependent on AEP cleavage of Tmod3, we constructed cell lines for use in functional assays (NC, AEP KD, AEP KD/tTmod3-C res, AEP KD/tTmod3-N res and AEP KD/tTmod3-N res/tTmod3-C res). Cells with AEP KD presented impaired proliferation and motility, which was consistent with our previous findings [36]. However, the rescue of tTmod3-C strikingly enhanced the proliferation and colony formation ability of U87-MG and A172 cells (Fig. 5A-B and Fig. S7A). The rescue of tTmod3-N significantly promoted the invasion of U87-MG and A172 cells in a Transwell assay (Fig. 5C-D).

To fully address the effects of AEP cleavage of Tmod3 on GBM progression in vivo, nude mice orthotopic glioma model using the constructed U87-MG cells (NC, AEP KD, AEP KD/tTmod3-C res, AEP KD/tTmod3-N res and AEP KD/tTmod3-N res/tTmod3-C res) were established. The MRI scan showed that AEP KD significantly inhibited tumorigenic ability of U87-MG cells, which was consistent with our previous article on AEP in GBM [36]. However, tTmod3-N and tTmod3-C, especially the latter one rescued the tumor size reduction caused by AEP KD. Notably, tTmod3-N together with tTmod3-C greatly promoted tumor progression in vivo (Fig. 5E-G). Mice injected with AEP-KD/tTmod3-C res or AEP-KD/tTmod3-N res/tTmod3-C res U87-MG cells presented with greater significant weight loss on the day of MRI detection (Fig. 5H). We calculated the OS of the remaining mice. Consistently, AEP KD significantly prolonged the survival of the mice; however, tTmod3-C res alone or in combination with tTmod3-N res dramatically shortened the OS of the related mice (Fig. 5I). Further measurement of the corresponding proliferation and invasion indicators by immunohistochemical analysis showed that Ki-67 was upregulated in tissues with tTmod3-C res, while Vinculin was upregulated in tissues with tTmod3-N res (Fig. S7B-D).

In order to exclude the excessive rescue of the two truncations of Tmod3 from producing side effects on cellular functions, we constructed GBM cells that were individually or collectively rescued with two truncations of Tmod3 at the physiological level (Fig. S8A). And we also detected and confirmed that the rescue of truncated Tmod3 does not have a significant effect on the known substrate protein p53 of AEP (Fig. S8A). Results of cellular functional assays consistently showed that truncated Tmod3 mediates the AEP-enhanced aggressive phenotype of cancer cells (Fig. S8B-D). In addition, the rescue of truncated Tmod3 had a limited effect on lysosomes (Fig. S9).

Taken together, the in vitro and in vivo experiments revealed that truncated Tmod3 produced by AEP promoted GBM progression. In particular, the proliferation-promoting ability of tTmod3-C led to a rapid increase in tumors in a short period of time, which directly affected the OS of model mice, while tTmod3-N significantly enhanced cancer cell migration and invasion.

### tTmod3-N promotes cancer cell invasion through actin remodeling.

**Fig. 6:**
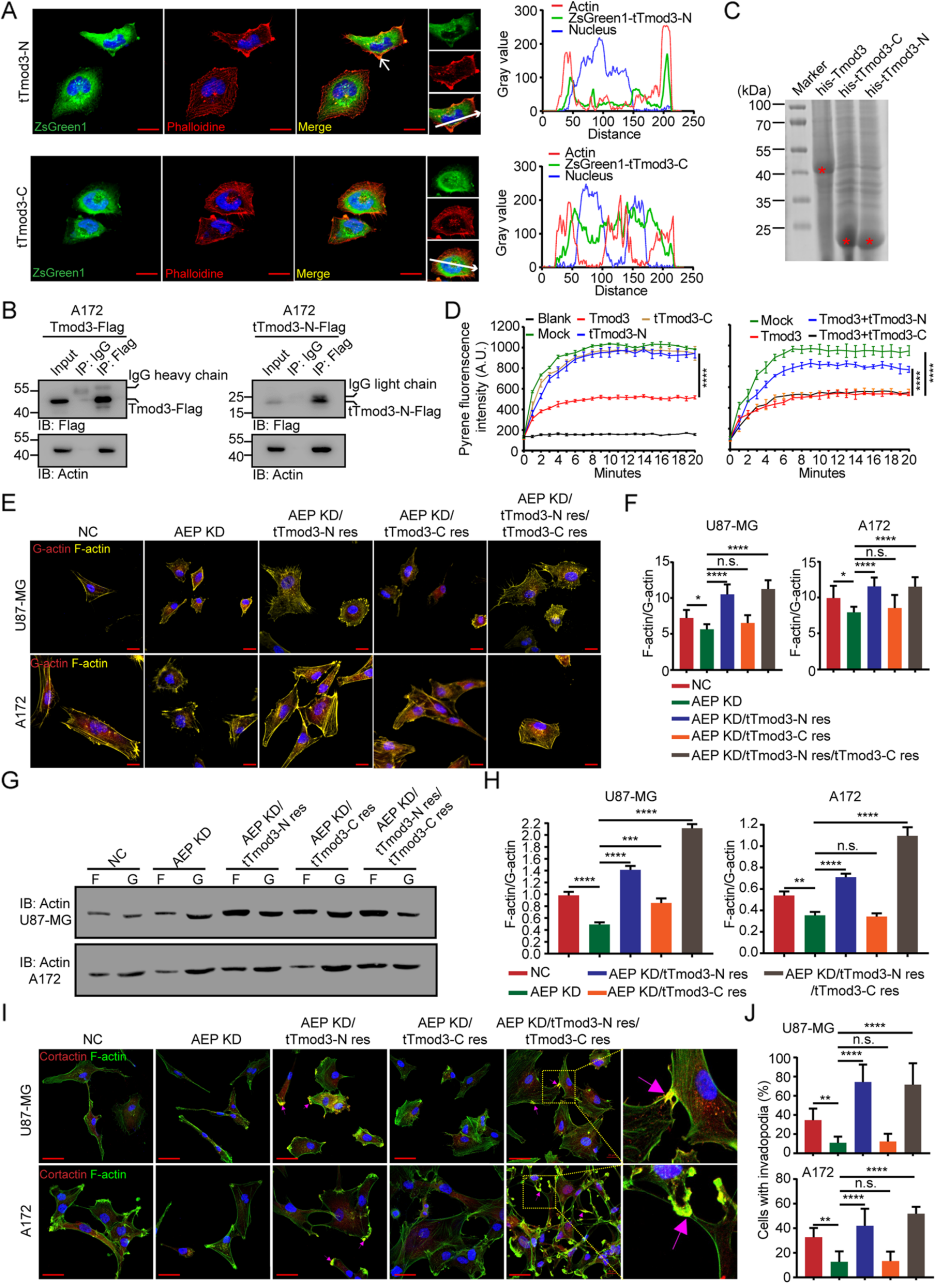
tTmod3-N promotes cancer cell invasion by actin remodeling.** A** Confocal images of A172 cells overexpressing ZsGreen1-tagged tTmod3-N or tTmod3-C (left panel) and co-localization tracer profiles along the line (white arrows) were indicated (right panel). Red, F-actin stained with phalloidine. Scale bar, 20 μm. **B** Co-immunoprecipitation assays for the interaction of Tmod3 or tTmod3-N with actin in A172 cells with Tmod3-Flag or tTmod3-N-Flag expression. **C** Lysates of prokaryotic expression of his-Tmod3, his-tTmod3-C and his-tTmod3-N in bacteria were stained with Coomassie brilliant blue R250. Red asterisks mark the targeted proteins. **D** Actin polymerization stimulated by Tmod3 or truncations of Tmod3 (200 nM). Actin polymerization was measured using Actin Polymerization Biochem Kit (BK003). **E** Representative fluorescent staining images of F-actin and G-actin in the indicated group of U87-MG and A172 cells. Yellow, F-actin; Red, G-actin. Scale bar, 20 μm. **F** Statistics of the F-actin/G-actin ratio of the indicated groups of U87-MG and A172 cells. Fluorescence intensity is captured by Image J. Fifty cells were randomly selected from each group for calculation. **G** Western blotting for F-actin and G-actin in U87-MG and A172 cells following cellular fractionation. The blots are representative of n = 3 independent experiments. **H** Statistics of the F-actin/G-actin ratio in the indicated groups of U87-MG and A172 cells. **I** Representative immunofluorescent staining of Cortactin and phalloidin staining of the indicated groups of U87-MG and A172 cells. Arrows indicate the co-localization of Cortactin with F-actin, which represents invadopodia. Scale bar, 40 μm. **J** Percentage of U87-MG and A172 cells with invadopodia. Five fields were randomly selected in each group. NC = negative control, cells expressing scramble shRNA. KD = knockdown, cells expressing AEP shRNA. ^*^P < 0.05, ^**^P < 0.01, ^***^P < 0.001, ^****^P < 0.0001, n.s. = no significance

Considering that Tmod3 is important for the regulation of the cytoskeleton, how do two truncations of Tmod3 contribute to cytoskeletal regulation? We used phalloidin staining to detect cytoskeletal changes in A172 cells with tTmod3-C or tTmod3-N overexpression. The cells overexpressing tTmod3-N tended to be disordered, with the cellular edges changing from sharp to rough and the disordered actin fibers were co-located with tTmod3-N at cell edges (Fig. 6A, upper panel). tTmod3-C was not closely related to actin, but was found translocated to the nucleus (Fig. 6A, bottom panel). Moreover, co-IP assay confirmed that both Tmod3 and tTmod3-N could bind to actin (Fig. 6B). Thus, these findings hinted that tTmod3-N may be involved in regulating the formation of actin fibers.

To investigate the effects of truncated Tmod3 on F-actin polymerization, we purified his-tagged full-length Tmod3 (Tmod3-FL), tTmod3-N and tTmod3-C (Fig. 6C). The rate of actin polymerization was detected after adding 200 mM Tmod3 or its truncations to the reaction system using an Actin Polymerization Biochem Kit. Indeed, Tmod3 markedly inhibited the polymerization of actin as previously reported [18], however, the two truncations alone failed to or mildly damage actin polymerization (Fig. 6D). When tTmod3-N combind with Tmod3-FL was added to the reaction system, the polymerization of actin was significantly rescued, which suggested that tTmod3-N may competitively inhibit the functions of Tmod3-FL and promote F-actin polymerization (Fig. 6D).

To further detect the effects of AEP cleavage of Tmod3 on actin remodeling. U87-MG and A172 cells with AEP KD or with truncated Tmod3 rescue and the related controls were analyzed (NC, AEP KD, AEP KD/tTmod3-N res, AEP KD/tTmod3-C res, AEP KD/tTmod3-N res/tTmod3-C res). Under normal conditions, globular-actin (G-actin) and F-actin are in dynamic equilibrium, and the F-actin to G-actin (F/G-actin) ratio can reflect the invasion ability of cells to some extent [45]. To test the changes in the F/G-actin ratio, IF staining of F-actin and G-actin with fluorescence-conjugated phalloidine and deoxyribonuclease I (Fig. 6E) and western blotting (Fig. 6G) were performed. The results indicated that AEP KD induced a decrease in the ratio of F/G-actin, while the rescue of tTmod3-N reversed this effect. The cells with tTmod3-N rescue appeared as extended pseudopodia and a disordered actin cytoskeleton. The rescue of tTmod3-C alone did not change the ratio of F/G-actin (Fig. 6F and Fig. 6H). Additionally, the cellular pseudopodia-related marker Cortactin was detected in related cells. In cells with the tTmod3-N res or tTmod3-N res/tTmod3-C res, Cortactin and F-actin were significantly colocalized at the edge of the cell, which was considered an invasive pseudopod [46] (Fig. 6I-J).

Hence, these findings demonstrated that tTmod3-N remodeled the actin cytoskeleton by interacting with actin and competitively suppressing full-length Tmod3 functions, which significantly promoted the invasion of cancer cells.

### tTmod3-C is accumulated in the nucleus and enhances SND1-mediated cancer cell proliferation.

**Fig. 7:**
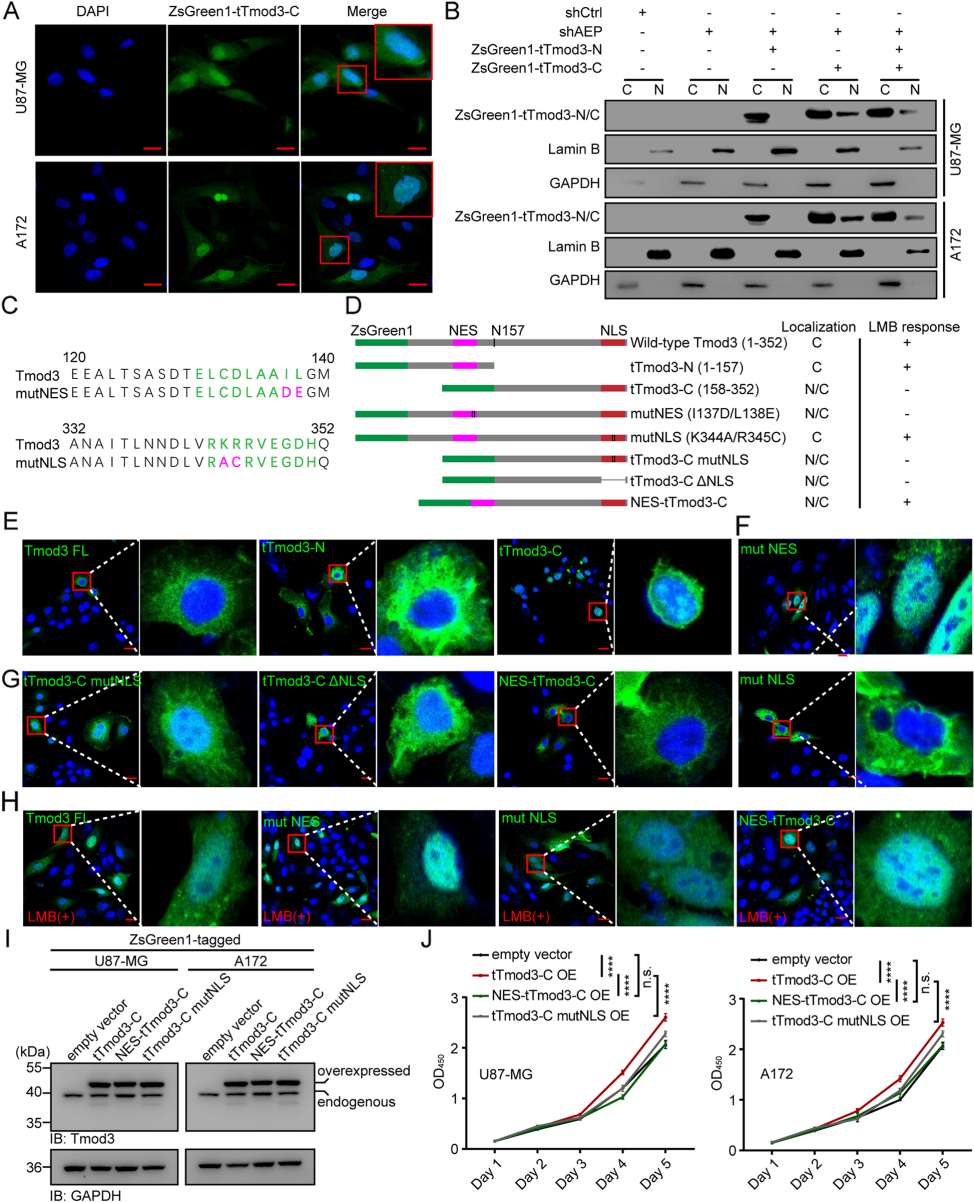
tTmod3-C is accumulated in the nucleus due to its NES sequence loss mediated by AEP cleavage.** A** Representative images of sub-localization of ZsGreen1-tagged tTmod3-C in A172 and U87-MG cells. Scale bars, 20 μm. **B** Immunoblots of ZsGreen1-tagged truncations of Tmod3, Lamin B and GAPDH in the indicated A172 and U87-MG cells following sub-cellular fractionation. C, cytoplasm; N, nucleus. **C** Putative NES and NLS identified in previous work. Conserved amino acids forming the putative NES or NLS are shown in green letters, and the amino acids that were changed by mutagenesis are in purple letters. **D** A schematic drawing of full-length Tmod3 and its truncations. Summarize their localization in HeLa cells in the absence and presence of Leptomycin B (LMB). **E** Representative images showing the sub-localization of full-length Tmod3, tTmod3-N and tTmod3-C in HeLa. **F** Representative images showing the sub-localization of NES or NLS mutant Tmod3 in HeLa cells. **G** Representative images showing the sub-localization of tTmod3-C with NLS mutation or deletion or NES addition in HeLa cells. **H** Representative images showing the sub-localization of full-length Tmod3, Tmod3 mutNES, Tmod3 mutNLS and NES-tTmod3-C in HeLa cells treated with LMB. Images are representative of n = 3 independent experiments. Scale bars, 20 μm. **I** Immunoblots of Tmod3 and GAPDH in the indicated U87-MG and A172cells with ZsGreen1 (empty vector), ZsGreen1-tagged tTmod3-C or its mutants overexpressed. **J** CCK-8 assay of the indicated U87-MG and A172 cells with ZsGreen1 (empty vector), ZsGreen1-tagged tTmod3-C, ZsGreen1-tagged NES-tTmod3-C or ZsGreen1-tagged tTmod3-C-mutantNLS overexpression. OE = overexpression. ^****^P < 0.0001, n.s. = no significance

tTmod3-C was found to aggregate in the nucleus of A172 and U87-MG cells by confocal laser scanning microscopy (Fig. 7A), which was also confirmed by western blotting (Fig. 7B). Tmod1, previously known as erythrocyte tropomodulin (E-Tmod), was transported to the nucleus depending on its functional nuclear import and export domains, and in the nucleus, it played a novel role in the proliferation of cells [47]. Inspired by this, we next set out to determine whether the nuclear export sequence (NES) (speculated to be located at aa__130-139_) or nuclear localization sequence (NLS) (speculated to be located at aa__343-351_) of Tmod3 is critical for the nuclear translocation of Tmod3. We transiently expressed Tmod3 or its truncations with NES or NLS mutations or deficiency in HeLa cells (Fig. 7C-D). Consistent with the results in the A172 and U87-MG cells, Tmod3 was mainly distributed in the cytoplasm, tTmod3-N was negligible in the nucleus, and tTmod3-C was obviously enriched in the nucleus (Fig. 7E). Mutations in the core amino acids of the NES sequence (I137D/L138E) led to nuclear accumulation of Tmod3, while mutations in the core amino acids of the NLS sequence (K344A/R345C) resulted in total cytoplasmic localization. These results indicated that both the NES and NLS contributed to the nuclear-cytoplasmic shuttling of Tmod3 (Fig. 7F). To determine whether the shuttling of tTmod3-C into the nucleus is dominantly mediated by the NLS or caused by NES deletion. We constructed tTmod3-C with an NLS mutation (tTmod3-C mutNLS) or deletion (tTmod3-C ΔNLS) and tTmod3-C with an NES addition (NES-tTmod3-C) (Fig. 7D). HeLa cells transiently expressing tTmod3-C mutNLS or tTmod3-C ΔNLS exhibited partial nuclear localization of the mutants, while most of the green fluorescence in cells that transiently expressed NES-tTmod3-C was in the cytoplasm (Fig. 7G). By the way, we treated HeLa cells transfected with the abovementioned truncations with leptomycin B (LMB, 100 nM, 3 h), a nuclear export inhibitor that inhibits the transport of proteins and RNAs carrying the NES. Indeed, truncations containing NES clearly responsed to LMB treatment (Fig. 7H). Defective NLS does not completely inhibit the nuclear aggregation of tTmod3-C. Functionally, the NES sequence addition can inhibit cell proliferation caused by tTmod3-C, while NLS mutated tTmod3-C still has a promotive effect on the proliferation of GBM cells (Fig. 7I-J). These results suggested that NES deficiency dominantly mediated the nuclear translocation of tTmod3-C, and AEP cleavage of Tmod3 at N157 was a natural regulatory mechanism of Tmod3 nuclear translocation because it cleaved the NES in the N-terminus.

**Fig. 8:**
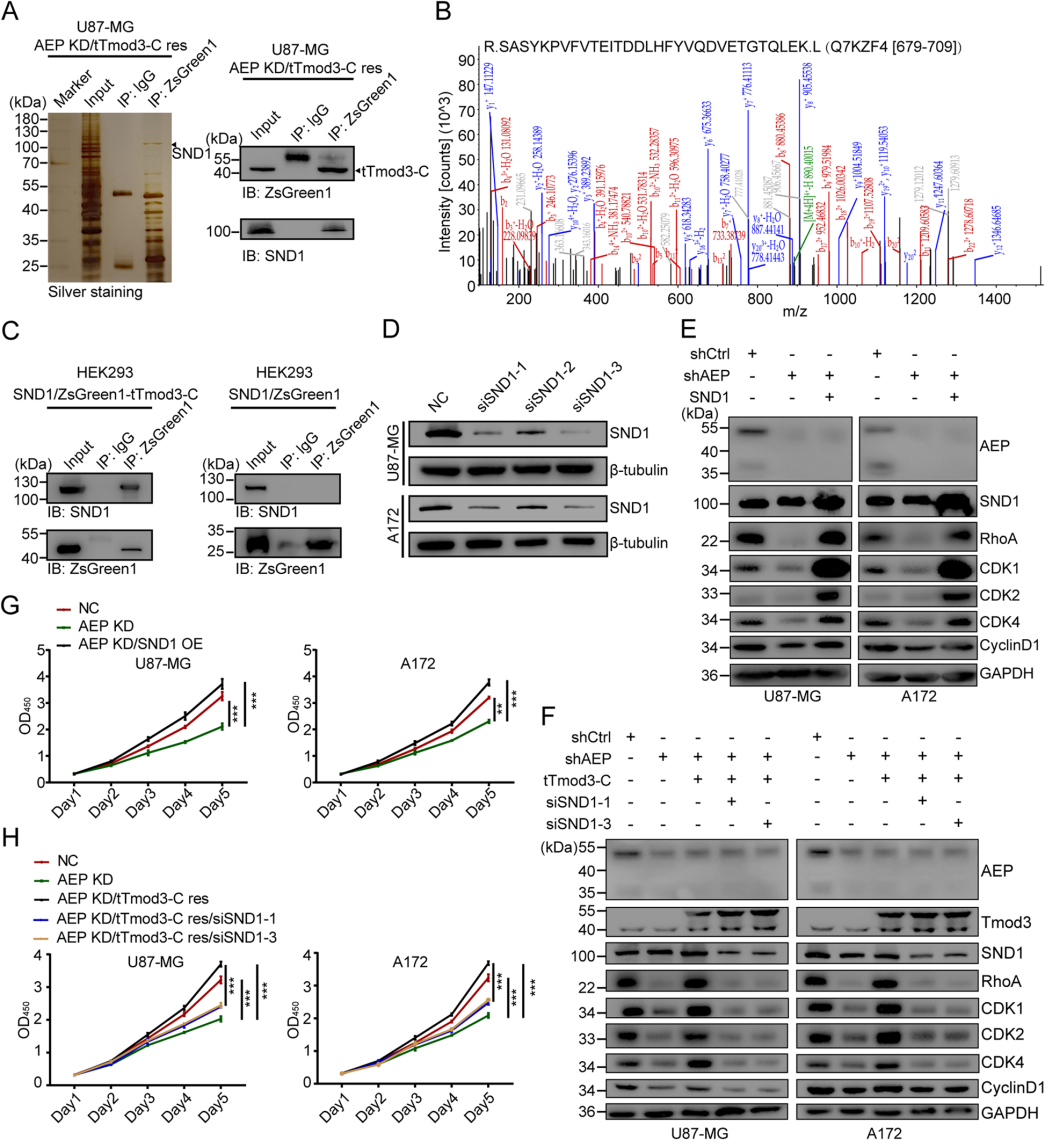
Nuclear tTmod3-C interacting with SND1 facilitates the growth of GBM by the RhoA/CDKs pathway.** A** Silver-stained gel showing immunoprecipitated ZsGreen1-tagged tTmod3-C or IgG and their bound proteins from U87-MG cells (Left panel); Immunoblot showing SND1 exist in anti-ZsGreen1-tTmod3-C immunoprecipitants (right panel). **B** Mass spectrometry analysis of proteins interacting with ZsGreen1-tagged tTmod3-C. The detected MS/MS peptide spectra of SND1 are shown. **C** Co-immunoprecipitation assays for the interaction of SND1 and ZsGreen1 or ZsGreen1-tagged tTmod3-C in HEK293 cells. **D** Immunoblots of SND1 in U87-MG and A172 cells treated with SND1 siRNA or siNC. **E** Immunoblots of the SND1/RhoA/CDKs axis in U87-MG and A172 with NC, AEP KD and AEP KD/SND1 OE. **F** Immunoblots of the SND1/RhoA/CDKs axis in U87-MG and A172 cells with NC, AEP KD, AEP KD/tTmod3-C res, AEP KD/tTmod3-C res/siSND1-1 and AEP KD/tTmod3-C res/siSND1-3. **G** CCK-8 assay of U87-MG and A172 cells with NC, AEP KD and AEP KD/SND1 OE. **H** CCK-8 assay of U87-MG and A172 cells with NC, AEP KD, AEP KD/tTmod3-C res, AEP KD/tTmod3-C res/siSND1-1 and AEP KD/tTmod3-C res/siSND1-3. NC = negative control, cells expressing scramble shRNA or siRNA. KD = knockdown, cells expressing AEP shRNA. ^**^P < 0.01, ^***^P < 0.001

To further address the mechanism by which tTmod3-C promotes cancer cell proliferation, mass spectrometry following co-IP using nuclear fractions of U87-MG cells with AEP KD and tTmod3-C rescue was conducted. The results revealed that Staphylococcal Nuclease And Tudor Domain Containing 1 (SND1) was a candidate for interacting with tTmod3-C (Fig. 8A-B, Supplementary Table 5). The interaction of tTmod3-C with SND1 was confirmed by co-IP with transiently expressed Flag-tagged SND1 and ZsGreen1-tTmod3-C in HEK293 cells followed by immunoblotting with anti-ZsGreen1 antibody (Fig. 8C).

SND1 is a novel transcriptional coactivator that activates downstream Ras Homolog Family Member A (RhoA) transcription, sequentially regulates expression of Cyclin D1 (CCND1), Cyclin E1 (CCNE1) and Cyclin Dependent Kinase 4 (CDK4), and accelerates cell proliferation in GBM [48, 49]. Together with *LGMN* and *TMOD3*, members of SNA1/RhoA signaling such as *SND1*, *RHOA* and *CDK1* were upregulated in GBM (Fig. S10A). We reasonably assumed that tTmod3-C promoted GBM cell proliferation by interacting with SND1. To confirm this supposition, A172 and U87-MG cells with indicated treatment: NC, AEP KD, AEP KD/SND1 OE, AEP KD/tTmod3-C res, AEP KD/tTmod3-C res/siSND1, were used. The effect of SND1 interference was confirmed by western blotting (Fig. 8D). The reported SND1 downstream targets such as RhoA, CDK1, CDK2, CDK4 and Cyclin D1, were significantly downregulated by AEP KD, but rescued by SND1 OE (Fig. 8E). tTmod3-C rescued the downregulation of the related markers caused by AEP KD, but SND1 interference reduced the effect of tTmod3-C in A172 and U87-MG cells (Fig. 8F). Compared to cells with AEP KD, GBM cells with SND1 OE showed reestablished proliferation, as detected by CCK-8, similar to the proliferation of cells with tTmod3-C rescue (Fig. 8G). However, when SND1 was interfered by siRNA, the promoted proliferation driven by tTmod3-C rescue was significantly inhibited, thus indicating that tTmod3-C promoted GBM proliferation partially depending on SND1 (Fig. 8H). In addition, correlation analysis of *TMOD3* and *SND1* or *RHOA* expression by GEPIA indicated that Tmod3 is correlated with SND1/RhoA signaling (Fig. S10B). These findings indicated that tTmod3-C was translocated to the nucleus mainly due to the absence of the NES in the N-terminus. Nuclear tTmod3-C interacting with SND1 promoted the transcription of downstream genes, facilitating cell proliferation.

## Discussion

Protease-mediated restriction enzyme cleavage modification was once considered to be a protein degradation mechanism due to its irreversibility. However, accumulating evidence has suggested that more truncated proteins are involved in various physiological or pathological processes, suggesting that enzymatic cleavage is a universal posttranslational protein regulation [50]. AEP is one of the key proteins that plays a novel role in the regulation of enzymatic cleavage. More and more substrates of AEP have been discovered, and increasing evidence has confirmed that AEP cleavage of substrates is an important regulatory event, especially in neurodegenerative diseases and malignant tumors [36–39]. Herein, we identified AEP cleavage of Tmod3, which produced two functional truncations. These two truncations played completely different roles in promoting cancer than those exhibited by Tmod3 through actin remodeling and SND1/RhoA-mediated cell proliferation. Promising experimental results provided evidence that AEP is an important protease cleavage regulatory protein, highlighting the role of AEP as a key mediator in cancers.

To maintain homeostasis, living organisms possess a finely tuned regulation system. Through intricate upstream regulation, AEP plays a "micro-scissor" role in the fine-tuned regulation of proteins. It specifically cleaves substrate proteins and exerts specific effects by altering the function of the substrate protein or by releasing truncations with novel functions. Despite inspiring findings, we noticed that the AEP cleavage rate of Tmod3 in tissues ranged from 25.0% to 32.0%, which was inferior to the AEP cleavage of p53 [36]. These results suggested that there may be negotiated complicated regulatory links to cleave Tmod3. However, the exact stimulus has not been identified. In vitro experiments confirm that AEP has a multistep autoactivation process requiring an acidic pH “window” between 4.5 and 3.5 [51, 52]. However, the final maturation of AEP is most likely not autocatalytic, and it is thought to be mediated by other lysosomal proteases, the identity of which remains uncertain [51]. Age, traumatic brain injury, chronic oral administration of the neurotoxin rotenone, dopamine metabolite or norepinephrine metabolite are activators of AEP [37–39], [53, 54]. In addition, our precious previous experiments indicated that AEP can also be partially activated by oxygen or nutrition deprivation [33, 36]. Spatially, AEP and its substrates colocalize in a cellular mechanism instead of transient random interaction, which is another direction for studying the precise regulation of Tmod3 cleavage.

Many studies have focused on the cytoskeletal regulatory function of Tmod3. However, few studies have explained the mechanisms by which Tmod3 plays a cancer-promoting effect in tumors [27, 28]. Our work directly elucidates that the tumor-promoting effects of Tmod3 are mediated by the truncations induced by AEP. Therefore, the detection of specific cleavage of Tmod3 in clinical samples can be used as an important biomarker for evaluating tumor malignancy. In fact, there is no recognized biomarker that can reflect both the invasive and proliferative ability of tumors. Tmod3, the AEP substrate in this study, has this potential. The detection of Tmod3-specific cleavage bands can be used to diagnose the malignancy of tumors to a certain extent, which has significant molecular diagnostic and clinical guidance implications. Although targeted drugs or specific small-molecule inhibitors of AEP have yet to be entered into the clinic [55, 56], our research provides a more solid theoretical basis for targeted AEP treatment.

Aberrant activity of core cell proliferation mechanisms is seen in essentially all tumor types and represents the driving force of tumorigenesis [57]. With the clinical success of CDK4/6 inhibitors, it is becoming increasingly clear that targeting individual cell cycle components may be an effective anti-cancer strategy [57, 58]. A definite link between AEP and cell proliferation has been reported that the proportion of G2/M phase cells was significantly decreased in cells with AEP knockdown [59]. However, the exact mechanism by which AEP regulates the cell proliferation is poorly understood. The present study suggests that the cell proliferation regulation by AEP through Tmod3 truncation also provides a practical basis for therapies targeting tumor proliferation.

## Conclusion

**Fig. 9:**
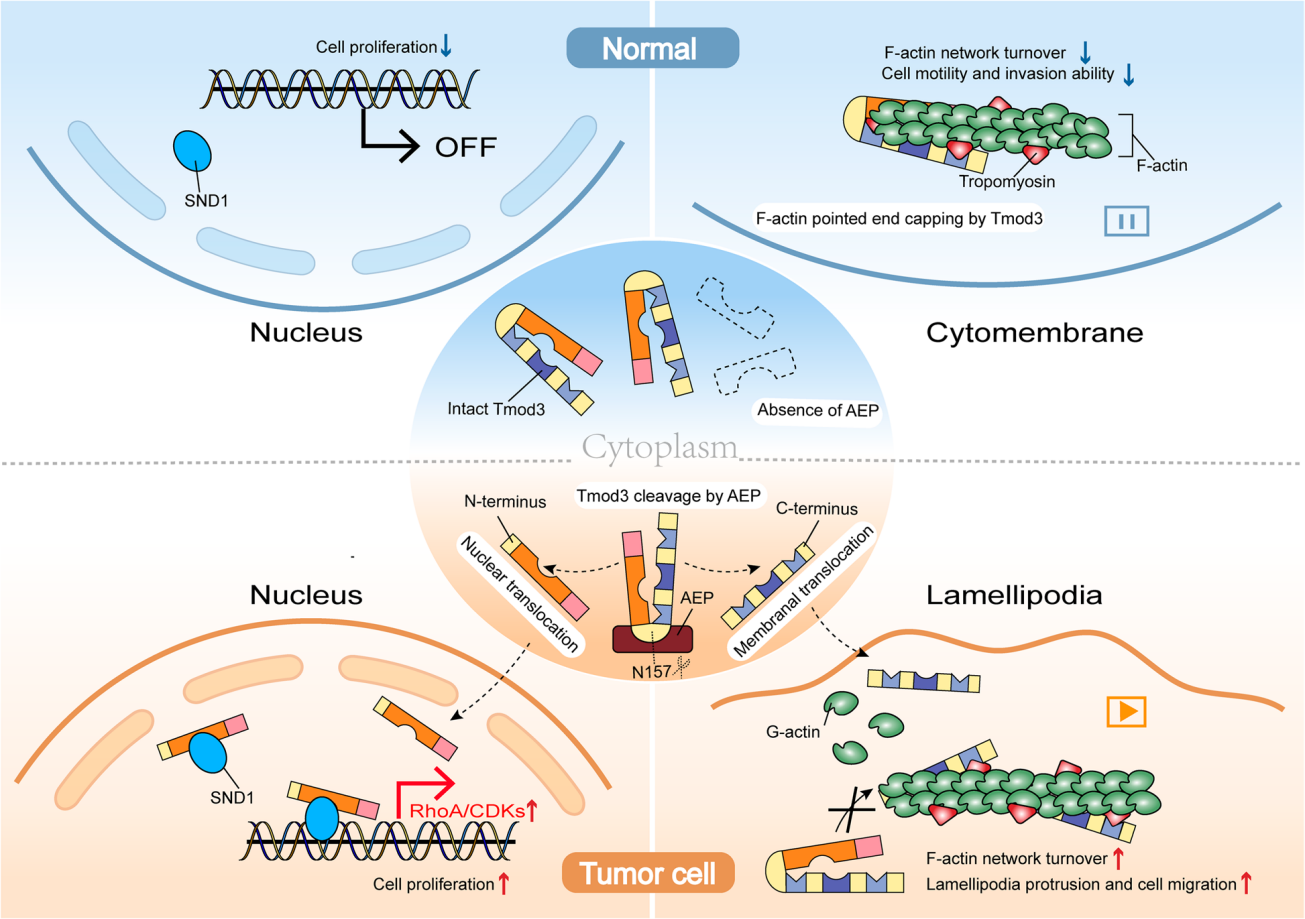
Graphical abstract. In the current study, we provide novel insights into the mechanisms underlying tumor progression through AEP cleavage of Tmod3. Mechanistically, tTmod3-N competitively inhibits the functions of Tmod3 in pointed-end capping to F-actin, promoting actin remodeling, thus reshaping the pseudopodia and promoting GBM invasion. tTmod3-C is translocated to the nucleus, where it interacts with SND1, resulting in RhoA/CDKs-mediated cell proliferation

In the current study, we provided vital insights into the mechanism underlying malignant cancer progression. We demonstrated that AEP-Tmod3 protease signaling axis played a “dual-regulation” role in promoting the rapid proliferation and malignant invasion of cancer cells in vitro and in vivo (Fig. 9). Truncations of Tmod3 induced by AEP contribute to cancer progression by facilitating actin remodeling and nuclear SND1 mediated RhoA/CDKs transcription**.**

## Supplementary Information

**Additional file 1: Fig. S1.** Relationship between AEP, Tmod3 and lysosome. **(A)** Representative images showing lysosomal status of A172 and U87-MG cells treated with shCtrl or shAEP (left panel) with quantification (right panel). Scale bar, 100 μm.** (B) **Representative confocal images showing the colocalization of AEP(red), Tmod3 (green) and LAMP2 (violet) in A172 cells by immunofluorescent staining (left panel, yellow arrows indicated the colocalization of AEP and Tmod3; white arrows indicated the colocalization of AEP, Tmod3 and LAMP2). Colocalization tracer profile along the line (white arrow)is indicated as merged image (right panel). Scale bar, 10 μm. NC=negative control, cells expressing scramble shRNA. KD=knockdown, cells expressing AEP shRNA.^*^P<0.05, ^**^P<0.01.

**Additional file 2: Fig. S2.** AEP binds to Tmod3 and specifically cleaves Tmod3 at N157.** (A)** Immunoblots of Flag-tagged Tmod3, AEP and GAPDH in HEK293 cells co-transfected with AEP and panels of Tmod3 point mutants. **(B)** Co-IP and western blotting analysis of interaction of Flag-tagged truncation of Tmod3 and AEP in HEK293 cells. **(C)** Pattern of a series of natural mutations in Tmod3. **(D)** Immunoblots of Flag-tagged Tmod3, AEP and GAPDH in HEK293 cells co-transfected with a series of Tmod3 mutants and AEP. **(E)**Immunoblots of Flag-tagged Tmod3 and GAPDH in HEK293 cells co-transfected with S71A- or S71D-mutant Tmod3 and AEP. **(F)**Immunoblots of Flag-tagged Tmod1, Tmod2 and Tmod4, AEP and GAPDH in HEK293 cells co-transfected with Tmods and AEP. WT=wild type.

**Additional file 3: Fig. S3.** Tmod3 is highly expressed in many solid tumors and associated with poor prognosis.** (A)** The relative expression of *TMOD3* in cholangiocarcinoma(CHOL), pancreatic adenocarcinoma (PAAD)and stomach adenocarcinoma(STAD) compared to normal tissues by GEPIA analysis. **(B)** Count of Tmod3 mRNA in glioma with different WHO grades in the GEO dataset GSE4290. **(C-G)** Log-rank analysis of OS for patients in the indicated groups with different Tmod3 expression levels in glioma (GBM and LGG), cervical squamous cellcarcinoma and endocervical adenocarcinoma (CESC), lung adenocarcinoma and lung squamous cellcarcinoma (LUAD and LUSC), pancreatic adenocarcinoma (PAAD) and bladder urothelial carcinoma (BLCA), as determined by GEPIA analysis. **(H)** Immunoblots of Tmod3 and GAPDH in fresh glioma or normal brain tissues. ^*^P<0.05, ^***^P<0.001, ^****^P<0.0001.

**Additional file 4:** **Fig. S4.** Suppression of Tmod3 inhibits tumor progression. **(A)** Representative images showing lysosomal status of U87-MG and A172 cells with or without Tmod3 KD (left panel) with quantification (right panel). Scale bar, 100 μm. **(B)**CCK-8 assays of U87-MG and A172 cells with or without Tmod3 KD. **(C, D)** Flow cytometry apoptosis analysis of U87-MG and A172 cells with or without Tmod3 KD. The data are presented as the mean ± s.d. **(E, F)** Scratch wound healing assay of U87-MG and A172 cells with or without Tmod3 KD. Triplicates in each group are the bases, the migration rate are presented as mean ± s.d. Scale bar, 200 μm.** (G)** Representative H&E images of each group are shown. Scale bar, 40 μm. **(H)** The weight of mice in each group on the day of MRI detection. The data are presented as the mean ± s.d. **(I)** Representative images showing IHC staining of Tmod3, Ki-67 and Vinculin in the indicated transplantation tumors of nude mice. Scale bar, 80 μm.**(J)** Relative expression of Tmod3,Ki-67 and Vinculin in the indicated groups of tumors. The data are presented as the mean ± s.d. NC=negative control, cells expressing scramble shRNA.KD=knockdown, cells expressing Tmod3 shRNA.^**^P<0.01, ^***^P<0.001,^****^P<0.0001, n.s.=no significance.

**Additional file 5: Fig. S5.** AEP produced Tmod3 truncations exist in many types of solid tumors. **(A, B) **Immunoblots of Tmod3, AEP and GAPDH in HGG tissues.**(C) **Immunoblots of Tmod3, AEP and GAPDH in LGG tissues. **(D)** Immunoblots of Tmod3, AEP and GAPDH in hepatocellular carcinoma tissues. **(E)**Immunoblots of Tmod3, AEP and GAPDH in cervical cancer tissues. Tissues with obvious cleavage of Tmod3 were marked in red.

**Additional file 6: Fig. S6.** The truncated Tmod3 produced by AEP promote GBM proliferation and invasion. **(A)** CCK-8 assays of U87-MG and A172 cells with or without truncations of Tmod3 overexpression. **(B)** Representative images showing IHC staining of Tmod3, Ki-67 and Vinculin in the indicated transplantation tumors in nude mice. Scale bars, 80 μm.**(C)** Relative expression of Tmod3, Ki-67 and Vinculin in the indicated groups of tumors. The data are presented as the mean ± s.d. NC=negative control, cells expressing ZsGreen1.OE=overexpression, cells expressing ZsGreen1-tTmod3-N or ZsGreen1-tTmod3-C.^*^P<0.05,^**^P<0.01,^***^P<0.001,^****^P<0.0001, n.s.=no significance.

**Additional file 7: Fig. S7.** AEP promotes GBM progression by cleaving Tmod3 *in vitro* and *in vivo*. **(A)** CCK-8 assays of U87-MG and A172 cells with NC, AEP KD, AEP KD/tTmod3-N res, AEP KD/tTmod3-C res and AEP KD/tTmod3-N res/tTmod3-C res. **(B)** Representative images showing IHC staining of Tmod3, Ki-67 and Vinculin in the indicated transplantation tumors in nude mice. Scale bar, 80 μm.(**C-D**) Relative expression of Ki-67,Tmod3 and Vinculin in the indicated groups. Data are presented as the mean ±s.d. NC=negative control, cells expressing scramble shRNA. KD=knockdown, cells expressing AEP shRNA.^*^P<0.05, ^**^P<0.01, ^****^P<0.0001, n.s.=no significance.

**Additional file 8: Fig. S8.** AEP promotes GBM progression by cleaving Tmod3*. *(A)Immunoblots of Tmod3, AEP, p53 and GAPDH in U87-MG and A172 cells with indicated treatments. (B) CCK-8 assays of U87-MG and A172 cells with NC, AEP KD, AEP KD/tTmod3-N-Flag res, AEPKD/tTmod3-C-Flag res and AEP KD/tTmod3-N-Flag res/tTmod3-C-Flag res. (C) Representative images showing colony formation assay of U87-MG and A172 cells with NC, AEP KD, AEP KD/tTmod3-N-Flag res, AEPKD/tTmod3-C-Flag res and AEP KD/tTmod3-N-Flag res/tTmod3-C-Flag res (left panel), with quantification (right panel). Scale bar, 100 μm. (D) Representative images showing Transwell assay of A172 andU87-MG cells with NC,AEP KD, AEP KD/tTmod3-N-Flag res, AEP KD/tTmod3-C-Flag res and AEPKD/tTmod3-N-Flag res/tTmod3-C-Flag res(left panel), with quantification (right panel). Scale bar, 100 μm. NC=negative control, cells expressing scramble shRNA. KD=knockdown, cells expressing AEP shRNA. ^*^P<0.05,^***^P<0.001, ^****^P<0.0001, n.s.=no significance.

**Additional file 9: Fig. S9.**Rescue of truncations of Tmod3 has limited effects on lysosomes in GBM cells.Representative images showing lysosomal status of indicated U87-MG and A172 cells (left panel) with quantification (right panel). Scale bar, 100 μm. NC=negative control, cells expressing scramble shRNA. KD=knockdown, cells expressing AEP shRNA.^*^P<0.05, n.s.=no significance.

**Additional file 10: Fig. S10.**Correlation analysis of Tmod3 and SND1/RhoA signaling.** (A) **The relative expression of *LGMN*, *TMOD3*, *ACTB*, *SND1*, *RHOA* and *CDK1* in GBM compared to normal brain tissues by GEPIA analysis. **(B) **Correlation analysis of *TMOD3* and *SND1* or *TMOD3* and *RHOA* expression in GBM by GEPIA analysis.^*^P<0.05.

**Additional file 11: Supplementary Table 1.** Primers and siRNAs.

**Additional file 12: Supplementary Table 2. **MS analysis of IP AEP.

**Additional file 13: Supplementary Table 3. **Tissue microarry and Tmod3 staining.

**Additional file 14: Supplementary Table 4.** Clinical characteristics of HGG patients.

**Additional file 15: Supplementary Table 5.** MS analysis of IP tTmod3-C.

**Additional file 16.** Supplementary materials and methods.

## Data Availability

All data used in this study are included within the article and additional files.

## Abbreviations

AEP: Asparagine endopeptidase
Tmod3: Tropomodulin-3
tTmod3-N: Truncated Tmod3 N terminal
tTmod3-C: Truncated Tmod3 C terminal
HGG: High-grade glioma
LGG: Low-grade glioma
F-actin: Filamentous-actin
G-actin: Globular-actin
SND1: Staphylococcal nuclease and tudor domain-containing 1
RhoA: Ras homolog family member A
CDKs: Cyclin dependent kinases
GBM: Glioblastoma multiforme
TM: Tropomyosin
ABS: Actin-binding site
LRR: Leucine-repeat regions
LGMN: Legumain
p53: Tumor protein p53
IF: Immunofluorescence
WT: Wild type
OE: Overexpression
KD: Knockdown
NC: Negative control
Akt2: AKT serine/threonine kinase 2
GEPIA: Gene Expression Profiling Interactive Analysis
CHOL: Cholangio carcinoma
PAAD: pancreatic adenocarcinoma
STAD: Stomach adenocarcinoma
GEO: Gene Expression Omnibus
CESC: Cervical squamous cell carcinoma and endocervical adenocarcinoma
LUAD: Lung adenocarcinoma
LUSC: Lung adenosquamous carcinoma
BLCA: Bladder urothelial carcinoma
snRNA-seq: Single-nuclear RNA-seq
scRNA-seq: Single-cell RNA-seq
t-SNE: T-distributed stochastic neighbor embedding
OS: Overall survival
WHO: World Health Organization
IDH1: Isocitrate dehydrogenase 1
CCK-8: Cell Counting Kit-8
Tmod3-FL: Full-length Tmod3
E-Tmod: Erythrocyte tropomodulin
NES: Nuclear export sequence
NLS: Nuclear localization sequence
LMB: Leptomycin B
