# Correction: Cleavage of tropomodulin-3 by asparagine endopeptidase promotes cancer malignancy by actin remodeling and SND1/RhoA signaling

**DOI:** 10.1186/s13046-022-02451-w

**Published:** 2022-08-04

**Authors:** Binghong Chen, Mengying Wang, Junjun Qiu, Keman Liao, Wenrui Zhang, Qi Lv, Chunhui Ma, Zhongrun Qian, Zhonggang Shi, Rong Liang, Yan Lin, Jiazhou Ye, Yongming Qiu, Yingying Lin

**Affiliations:** 1 Department of Neurosurgery, Brain Injury Center, Ren Ji Hospital, Shanghai Jiao Tong University School of Medicine, Shanghai, 200127 People’s Republic of China; 2 Department of Gynaecology, Obstetrics and Gynaecology Hospital, Fudan University, Shanghai, People’s Republic of China; 3 Department of Radiology, School of Medicine, Tongji Hospital, Tongji University, Shanghai, People’s Republic of China; 4 Department of Orthopedics, Shanghai General Hospital of Shanghai Jiao Tong University, Shanghai, People’s Republic of China; 5 Department of Neurosurgery, Division of Life Science and Medicine, The First Affiliated Hospital of University of Science and Technology of China, Hefei, Anhui People’s Republic of China; 6 Department of Medical Oncology, Guangxi Medical University Cancer Hospital, Nanning, Guangxi People’s Republic of China; 7 Department of Hepatobiliary Surgery, Guangxi Medical University Cancer Hospital, Nanning, Guangxi People’s Republic of China

**Correction: J Exp Clin Cancer Res 41, 209 (2022)**

**https://doi.org/10.1186/s13046-022-02411-4**

Following publication of the original article [1], an error was identified in Fig. 2; specifically:

**Fig. 2:**
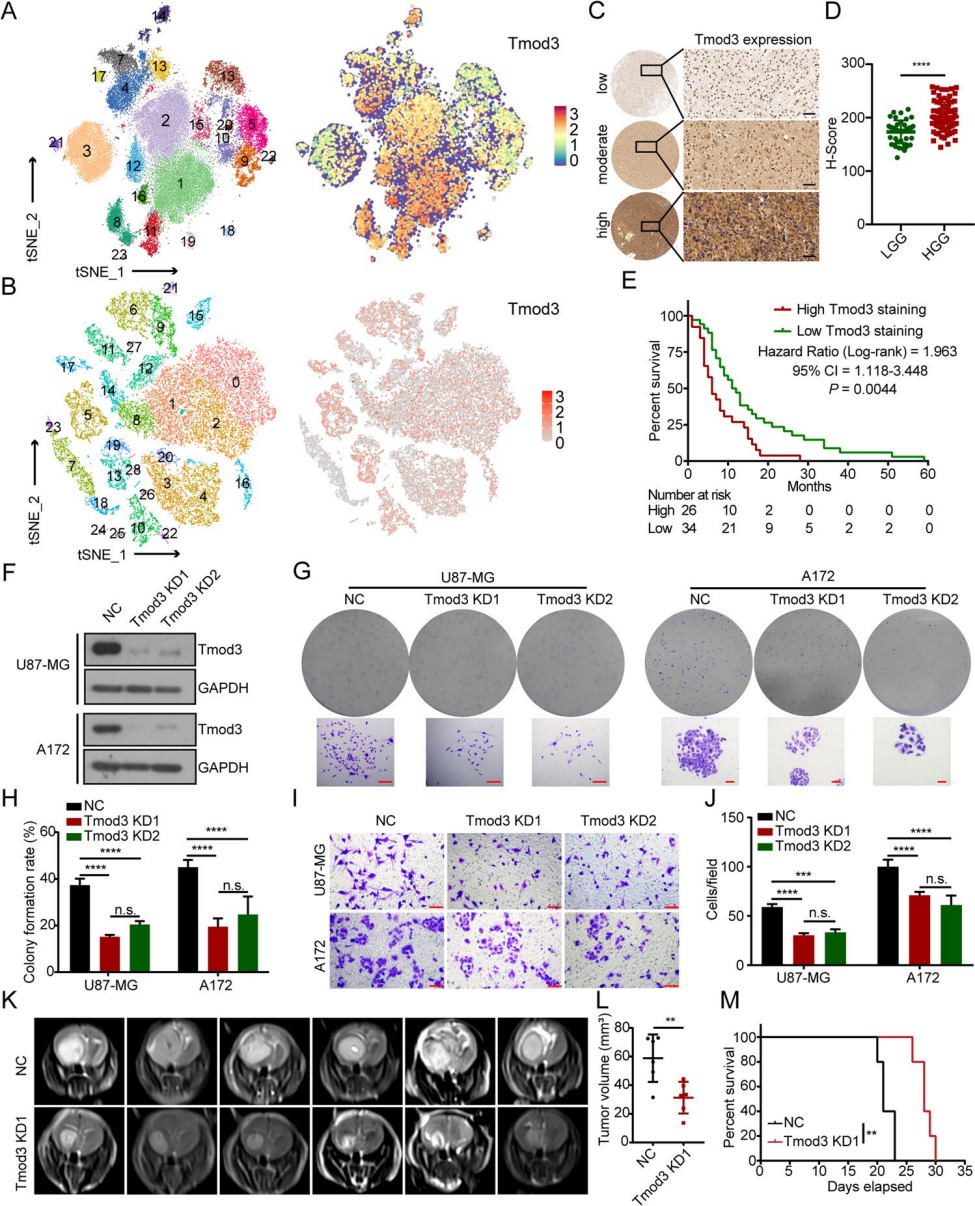
Tmod3 is highly expressed in many solid tumors and is associated with poor patient prognosis. **A**, **B** snRNA-seq with GBM cells (**A**) and scRNA-seq with cervical cancer cells (**B**) populations colored by clusters, number of cells analyzed in each cluster in the legend and the expression of Tmod3 in each cluster. The darker the red color, the higher the expression of the corresponding gene in that cell. **C** Representative images of low, moderate and high IHC staining of Tmod3 in glioma tissue array. Scale bar, 40 μm. **D** Histochemistry score (H-score) of Tmod3 in LGG and HGG of the tissue array; the data are shown as the mean ± s.d. **E** Kaplan–Meier survival plots showing survival rates of the patients in the indicated groups with high or low Tmod3 H-scores. Comparisons of survival curves were performed with Log-rank test. **F** Immunoblots of Tmod3 and GAPDH in U87-MG and A172 cells with or without Tmod3 KD. **G**, **H** Colony formation assay of U87-MG and A172 cells with or without Tmod3 KD. Scale bar, 100 μm. Statistical analysis of colony formation rate was done (H). Triplicates in each group are the bases for the presented mean ± s.d. **I**, **J** Transwell assay of U87-MG and A172 cells with or without Tmod3 KD. Scale bar, 100 μm. Triplicates in each group, data are presented as mean ± s.d. **K** Representative coronal MRI of xenograft GBM tumors orthotopically inoculated with U87-MG cells with or without Tmod3 KD on the 20th day postoperation (*n* = 8 per group). **L** The tumor volume was calculated in each group using the Coniglobus formula method. The data are presented as the mean ± s.d. **M** Kaplan–Meier plot analysis of the OS of mice in the indicated groups (*n* = 5 per group). Comparisons of survival curves were performed with Log-rank test. NC = negative control, cells expressing scramble shRNA. KD = knockdown, cells expressing Tmod3 shRNA. **P < 0.01, ***P < 0.001, ****P < 0.0001, n.s. = no significance

Figure 2 I: A172-NC was overlaid by U87-Tmod3-KD2; correct image is now used.

The correction does not have any effect on the results or conclusions of the paper. The original article has been corrected.
