# Supplementary figures and images for: Cleavage of tropomodulin-3 by asparagine endopeptidase promotes cancer malignancy by actin remodeling and SND1/RhoA signaling
Source: J Exp Clin Cancer Res. 2022 Jun 28;41:209. doi: 10.1186/s13046-022-02411-4 (PMC9238189; doi:10.1186/s13046-022-02411-4)

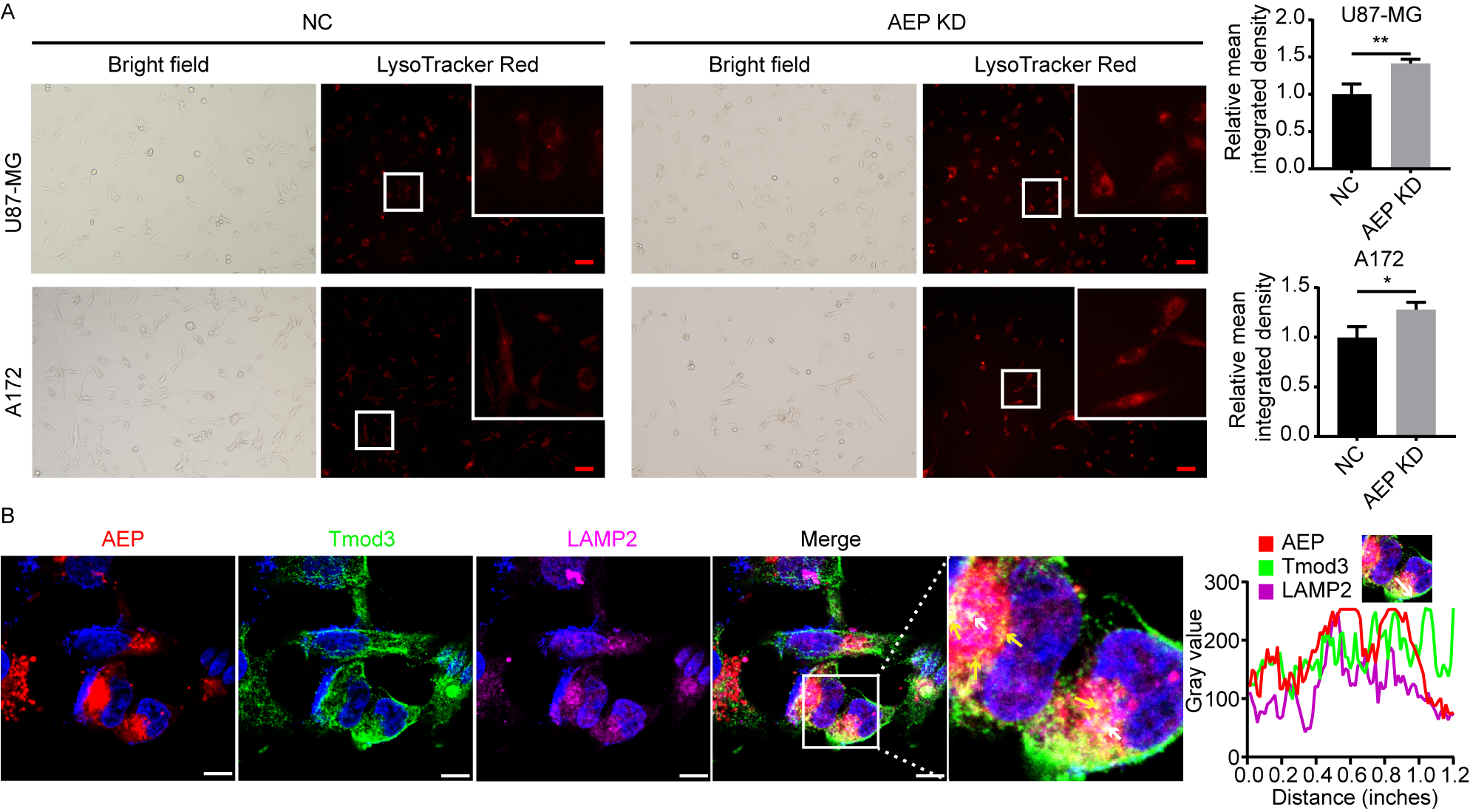

Supplement: Supplementary file 1 — Additional file 1: Fig. S1. Relationship between AEP, Tmod3 and lysosome. (A) Representative images showing lysosomal status of A172 and U87-MG cells treated with shCtrl or shAEP (left panel) with quantification (right panel). Scale bar, 100 μm. (B) Representative confocal images showing the colocalization of AEP(red), Tmod3 (green) and LAMP2 (violet) in A172 cells by immunofluorescent staining (left panel, yellow arrows indicated the colocalization of AEP and Tmod3; white arrows indicated the colocalization of AEP, Tmod3 and LAMP2). Colocalization tracer profile along the line (white arrow)is indicated as merged image (right panel). Scale bar, 10 μm. NC=negative control, cells expressing scramble shRNA. KD=knockdown, cells expressing AEP shRNA.*P<0.05, **P<0.01. [file 13046_2022_2411_MOESM1_ESM.tif]

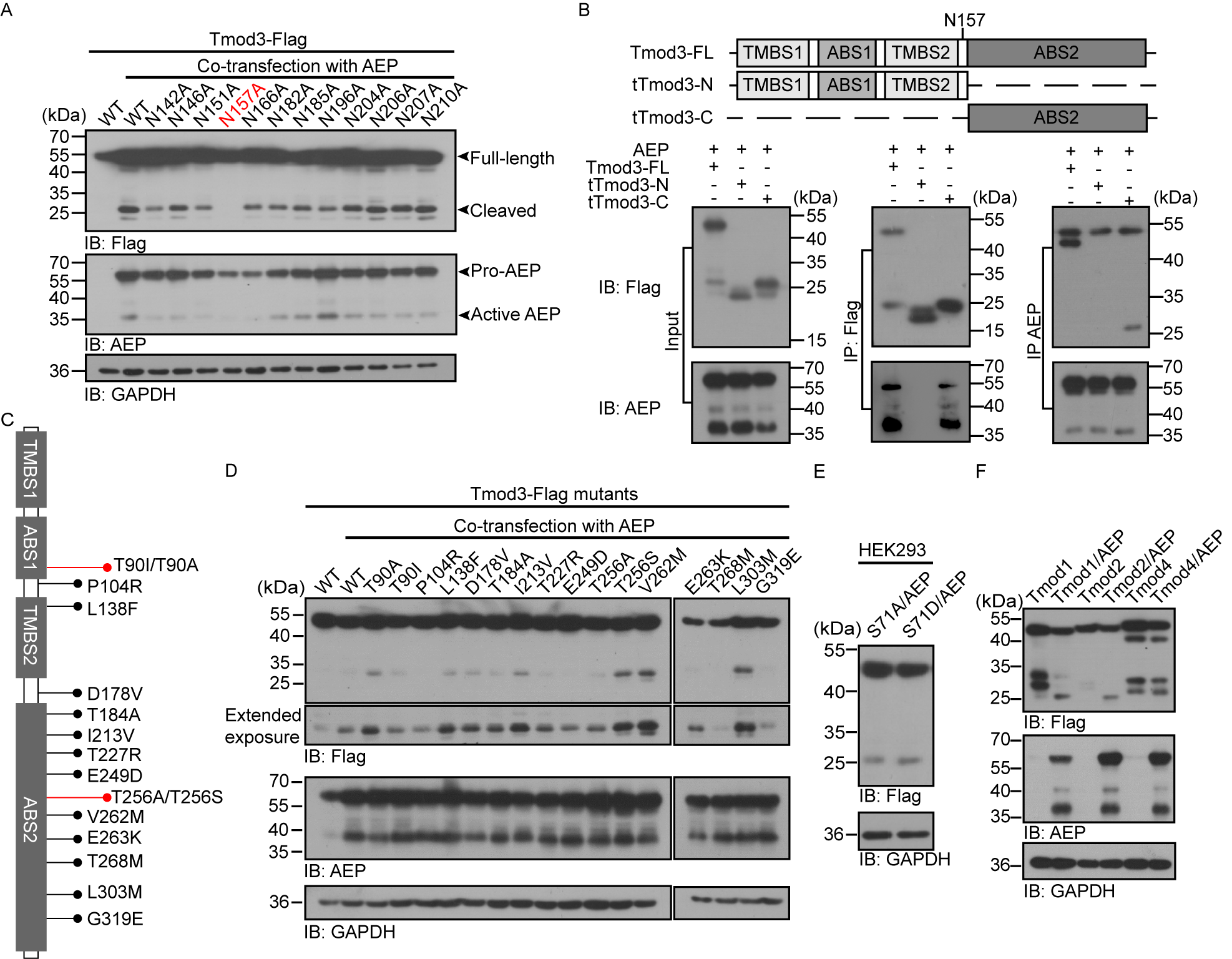

Supplement: Supplementary file 2 — Additional file 2: Fig. S2. AEP binds to Tmod3 and specifically cleaves Tmod3 at N157. (A) Immunoblots of Flag-tagged Tmod3, AEP and GAPDH in HEK293 cells co-transfected with AEP and panels of Tmod3 point mutants. (B) Co-IP and western blotting analysis of interaction of Flag-tagged truncation of Tmod3 and AEP in HEK293 cells. (C) Pattern of a series of natural mutations in Tmod3. (D) Immunoblots of Flag-tagged Tmod3, AEP and GAPDH in HEK293 cells co-transfected with a series of Tmod3 mutants and AEP. (E)Immunoblots of Flag-tagged Tmod3 and GAPDH in HEK293 cells co-transfected with S71A- or S71D-mutant Tmod3 and AEP. (F)Immunoblots of Flag-tagged Tmod1, Tmod2 and Tmod4, AEP and GAPDH in HEK293 cells co-transfected with Tmods and AEP. WT=wild type. [file 13046_2022_2411_MOESM2_ESM.tif]

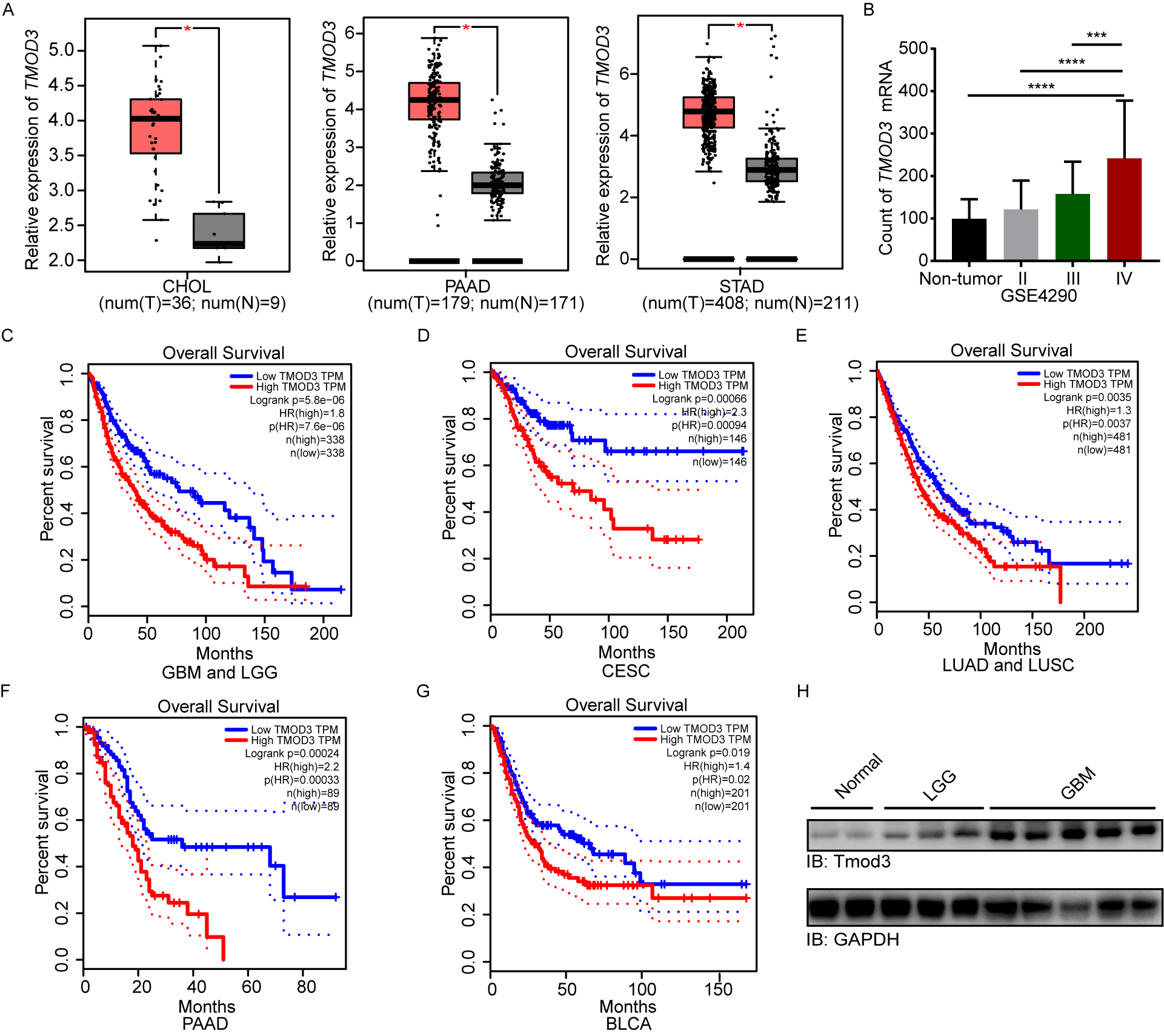

Supplement: Supplementary file 3 — Additional file 3: Fig. S3. Tmod3 is highly expressed in many solid tumors and associated with poor prognosis. (A) The relative expression of TMOD3 in cholangiocarcinoma(CHOL), pancreatic adenocarcinoma (PAAD)and stomach adenocarcinoma(STAD) compared to normal tissues by GEPIA analysis. (B) Count of Tmod3 mRNA in glioma with different WHO grades in the GEO dataset GSE4290. (C-G) Log-rank analysis of OS for patients in the indicated groups with different Tmod3 expression levels in glioma (GBM and LGG), cervical squamous cellcarcinoma and endocervical adenocarcinoma (CESC), lung adenocarcinoma and lung squamous cellcarcinoma (LUAD and LUSC), pancreatic adenocarcinoma (PAAD) and bladder urothelial carcinoma (BLCA), as determined by GEPIA analysis. (H) Immunoblots of Tmod3 and GAPDH in fresh glioma or normal brain tissues. *P<0.05, ***P<0.001, ****P<0.0001. [file 13046_2022_2411_MOESM3_ESM.tif]

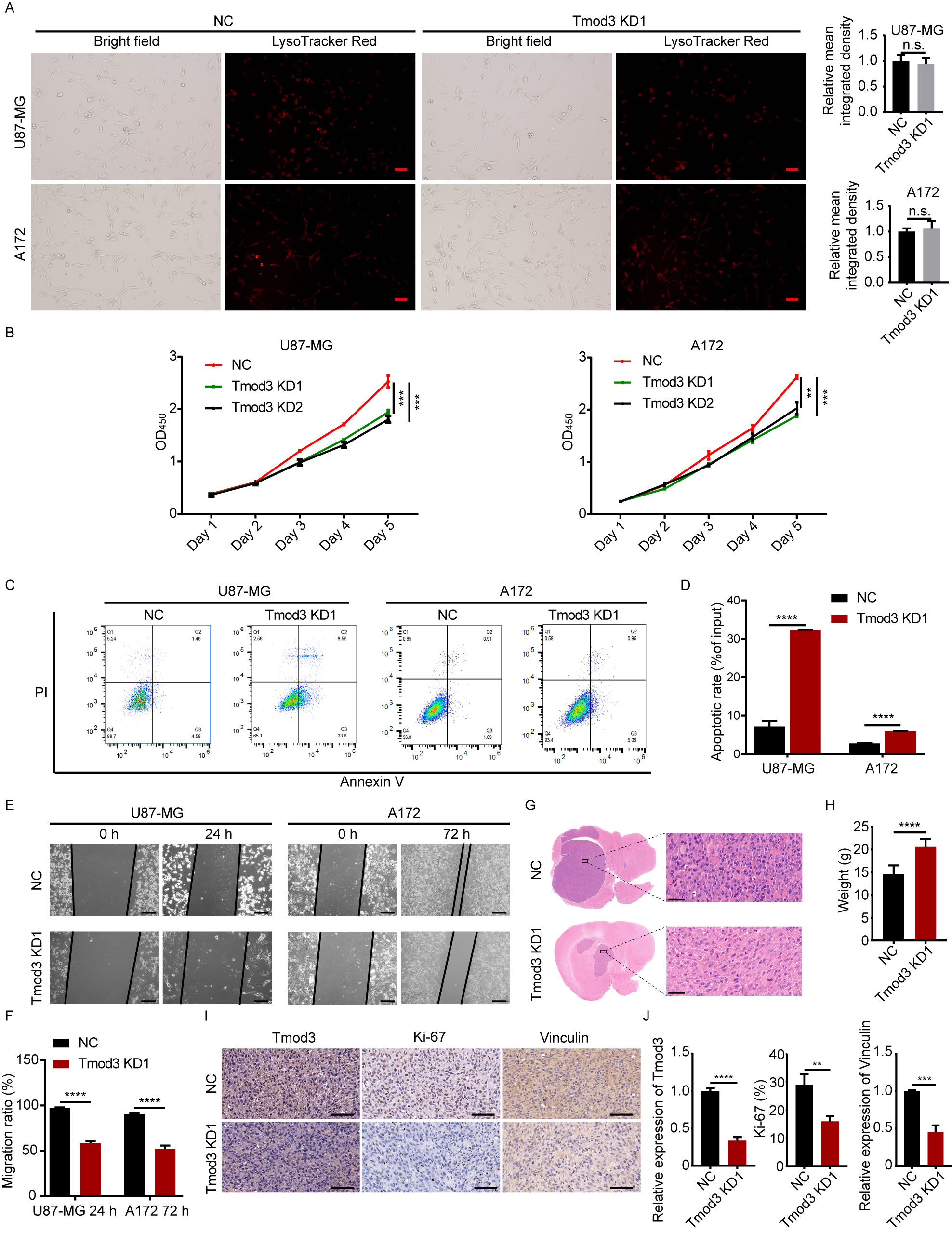

Supplement: Supplementary file 4 — Additional file 4: Fig. S4. Suppression of Tmod3 inhibits tumor progression. (A) Representative images showing lysosomal status of U87-MG and A172 cells with or without Tmod3 KD (left panel) with quantification (right panel). Scale bar, 100 μm. (B)CCK-8 assays of U87-MG and A172 cells with or without Tmod3 KD. (C, D) Flow cytometry apoptosis analysis of U87-MG and A172 cells with or without Tmod3 KD. The data are presented as the mean ± s.d. (E, F) Scratch wound healing assay of U87-MG and A172 cells with or without Tmod3 KD. Triplicates in each group are the bases, the migration rate are presented as mean ± s.d. Scale bar, 200 μm. (G) Representative H&E images of each group are shown. Scale bar, 40 μm. (H) The weight of mice in each group on the day of MRI detection. The data are presented as the mean ± s.d. (I) Representative images showing IHC staining of Tmod3, Ki-67 and Vinculin in the indicated transplantation tumors of nude mice. Scale bar, 80 μm.(J) Relative expression of Tmod3,Ki-67 and Vinculin in the indicated groups of tumors. The data are presented as the mean ± s.d. NC=negative control, cells expressing scramble shRNA.KD=knockdown, cells expressing Tmod3 shRNA.**P<0.01, ***P<0.001,****P<0.0001, n.s.=no significance. [file 13046_2022_2411_MOESM4_ESM.tif]

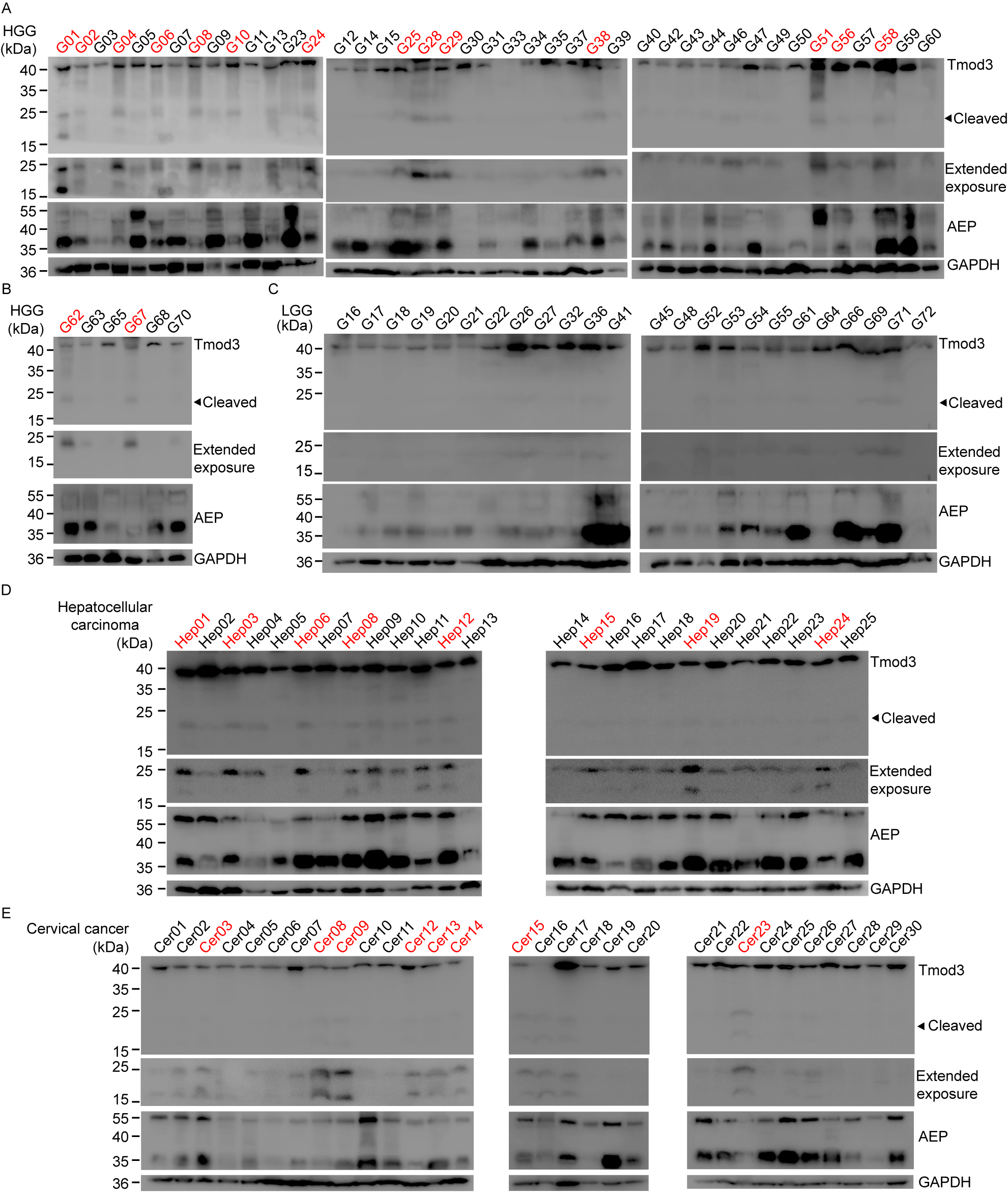

Supplement: Supplementary file 5 — Additional file 5: Fig. S5. AEP produced Tmod3 truncations exist in many types of solid tumors. (A, B) Immunoblots of Tmod3, AEP and GAPDH in HGG tissues.(C) Immunoblots of Tmod3, AEP and GAPDH in LGG tissues. (D) Immunoblots of Tmod3, AEP and GAPDH in hepatocellular carcinoma tissues. (E)Immunoblots of Tmod3, AEP and GAPDH in cervical cancer tissues. Tissues with obvious cleavage of Tmod3 were marked in red. [file 13046_2022_2411_MOESM5_ESM.tif]

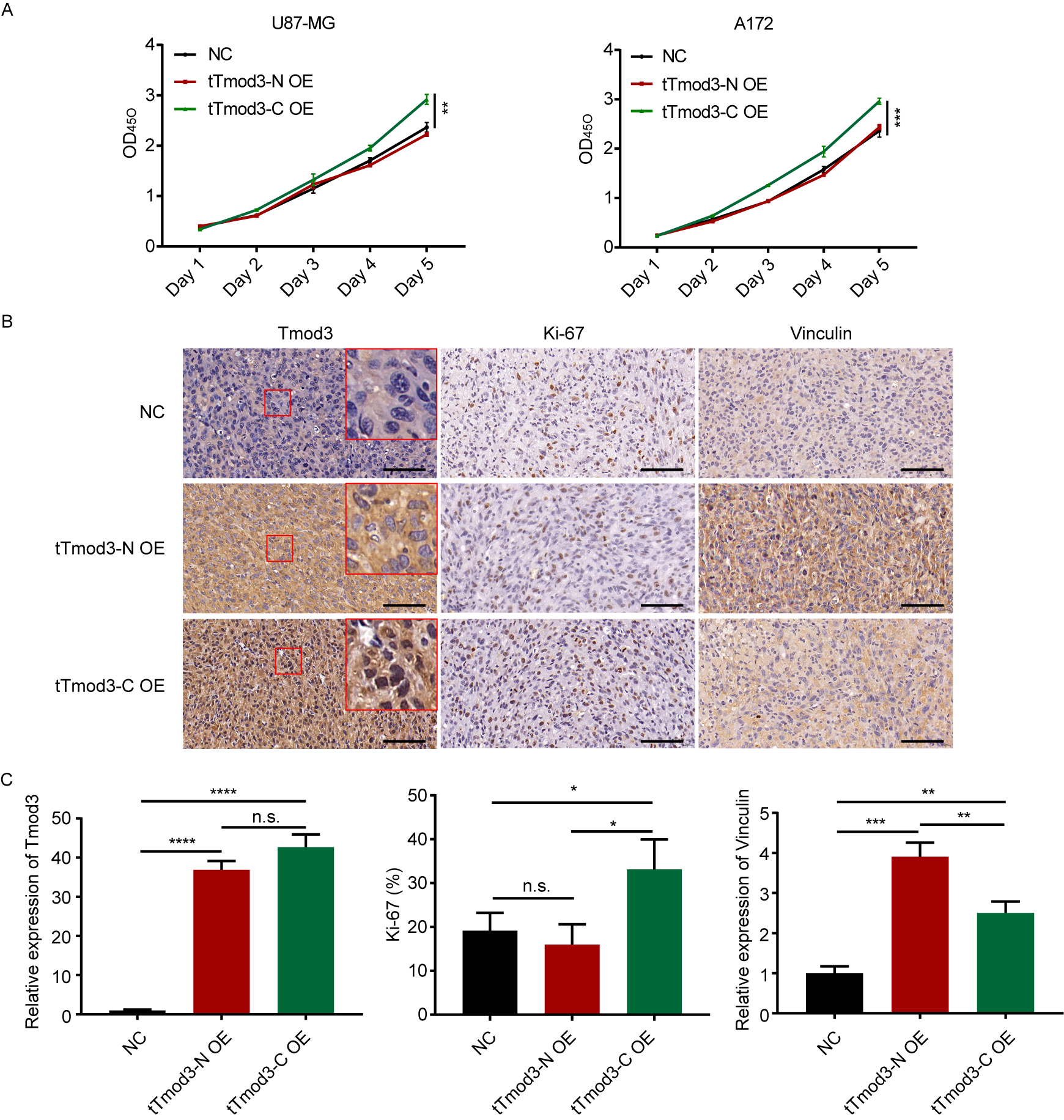

Supplement: Supplementary file 6 — Additional file 6: Fig. S6. The truncated Tmod3 produced by AEP promote GBM proliferation and invasion. (A) CCK-8 assays of U87-MG and A172 cells with or without truncations of Tmod3 overexpression. (B) Representative images showing IHC staining of Tmod3, Ki-67 and Vinculin in the indicated transplantation tumors in nude mice. Scale bars, 80 μm.(C) Relative expression of Tmod3, Ki-67 and Vinculin in the indicated groups of tumors. The data are presented as the mean ± s.d. NC=negative control, cells expressing ZsGreen1.OE=overexpression, cells expressing ZsGreen1-tTmod3-N or ZsGreen1-tTmod3-C.*P<0.05,**P<0.01,***P<0.001,****P<0.0001, n.s.=no significance. [file 13046_2022_2411_MOESM6_ESM.tif]

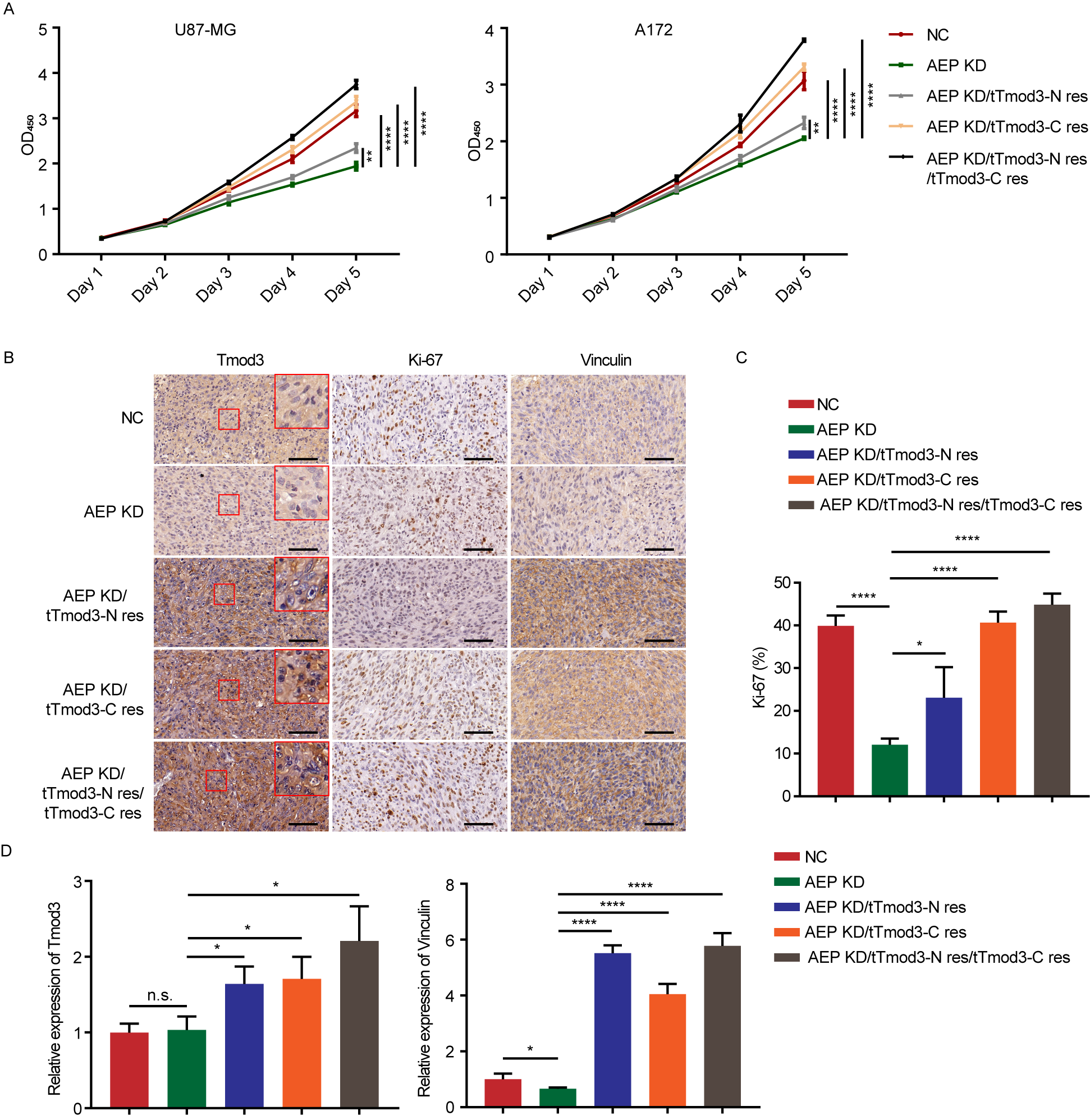

Supplement: Supplementary file 7 — Additional file 7: Fig. S7. AEP promotes GBM progression by cleaving Tmod3 in vitro and in vivo. (A) CCK-8 assays of U87-MG and A172 cells with NC, AEP KD, AEP KD/tTmod3-N res, AEP KD/tTmod3-C res and AEP KD/tTmod3-N res/tTmod3-C res. (B) Representative images showing IHC staining of Tmod3, Ki-67 and Vinculin in the indicated transplantation tumors in nude mice. Scale bar, 80 μm.(C-D) Relative expression of Ki-67,Tmod3 and Vinculin in the indicated groups. Data are presented as the mean ±s.d. NC=negative control, cells expressing scramble shRNA. KD=knockdown, cells expressing AEP shRNA.*P<0.05, **P<0.01, ****P<0.0001, n.s.=no significance. [file 13046_2022_2411_MOESM7_ESM.tif]

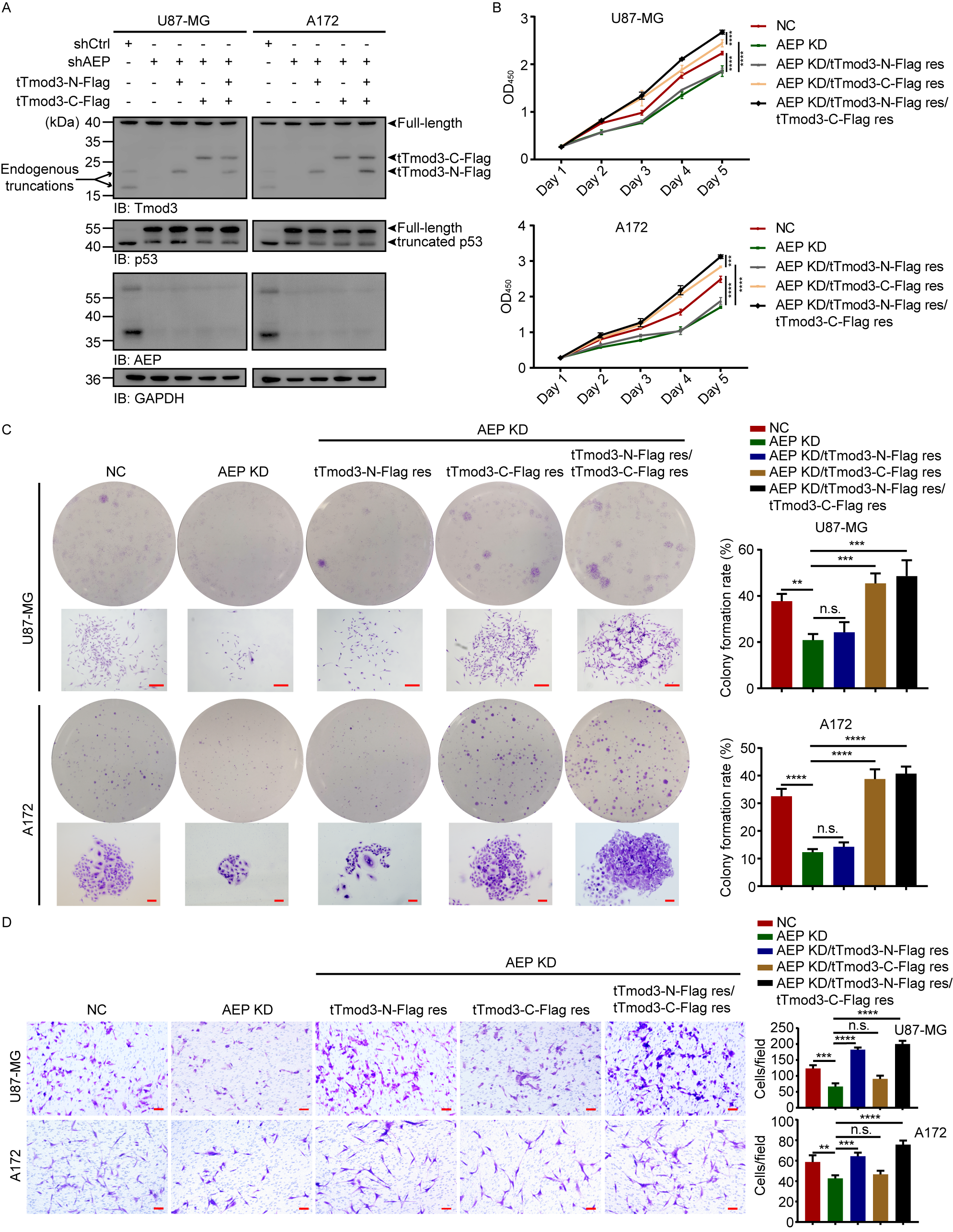

Supplement: Supplementary file 8 — Additional file 8: Fig. S8. AEP promotes GBM progression by cleaving Tmod3. (A)Immunoblots of Tmod3, AEP, p53 and GAPDH in U87-MG and A172 cells with indicated treatments. (B) CCK-8 assays of U87-MG and A172 cells with NC, AEP KD, AEP KD/tTmod3-N-Flag res, AEPKD/tTmod3-C-Flag res and AEP KD/tTmod3-N-Flag res/tTmod3-C-Flag res. (C) Representative images showing colony formation assay of U87-MG and A172 cells with NC, AEP KD, AEP KD/tTmod3-N-Flag res, AEPKD/tTmod3-C-Flag res and AEP KD/tTmod3-N-Flag res/tTmod3-C-Flag res (left panel), with quantification (right panel). Scale bar, 100 μm. (D) Representative images showing Transwell assay of A172 andU87-MG cells with NC,AEP KD, AEP KD/tTmod3-N-Flag res, AEP KD/tTmod3-C-Flag res and AEPKD/tTmod3-N-Flag res/tTmod3-C-Flag res(left panel), with quantification (right panel). Scale bar, 100 μm. NC=negative control, cells expressing scramble shRNA. KD=knockdown, cells expressing AEP shRNA. *P<0.05,***P<0.001, ****P<0.0001, n.s.=no significance. [file 13046_2022_2411_MOESM8_ESM.tif]

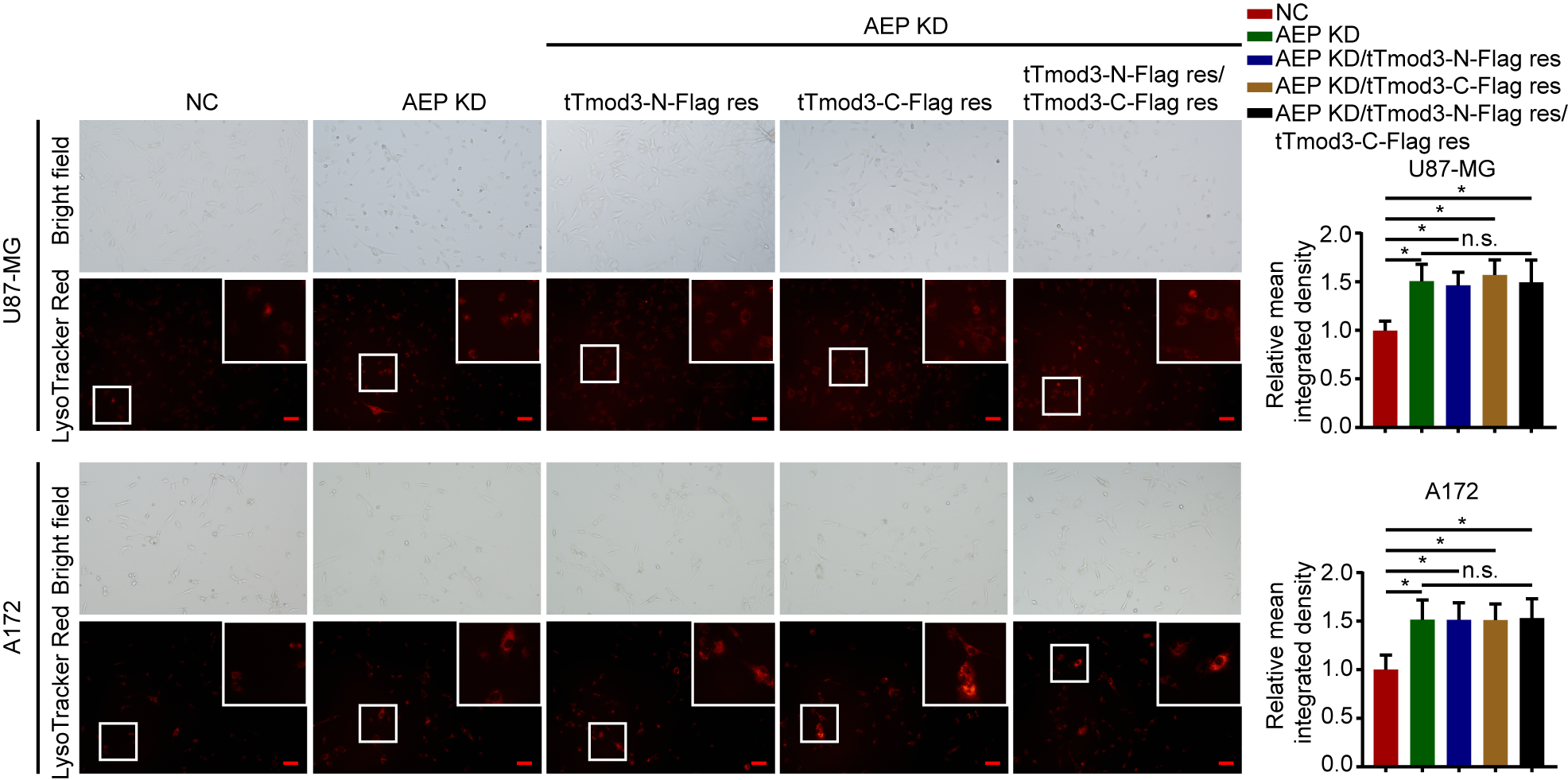

Supplement: Supplementary file 9 — Additional file 9: Fig. S9.Rescue of truncations of Tmod3 has limited effects on lysosomes in GBM cells.Representative images showing lysosomal status of indicated U87-MG and A172 cells (left panel) with quantification (right panel). Scale bar, 100 μm. NC=negative control, cells expressing scramble shRNA. KD=knockdown, cells expressing AEP shRNA.*P<0.05, n.s.=no significance. [file 13046_2022_2411_MOESM9_ESM.tif]

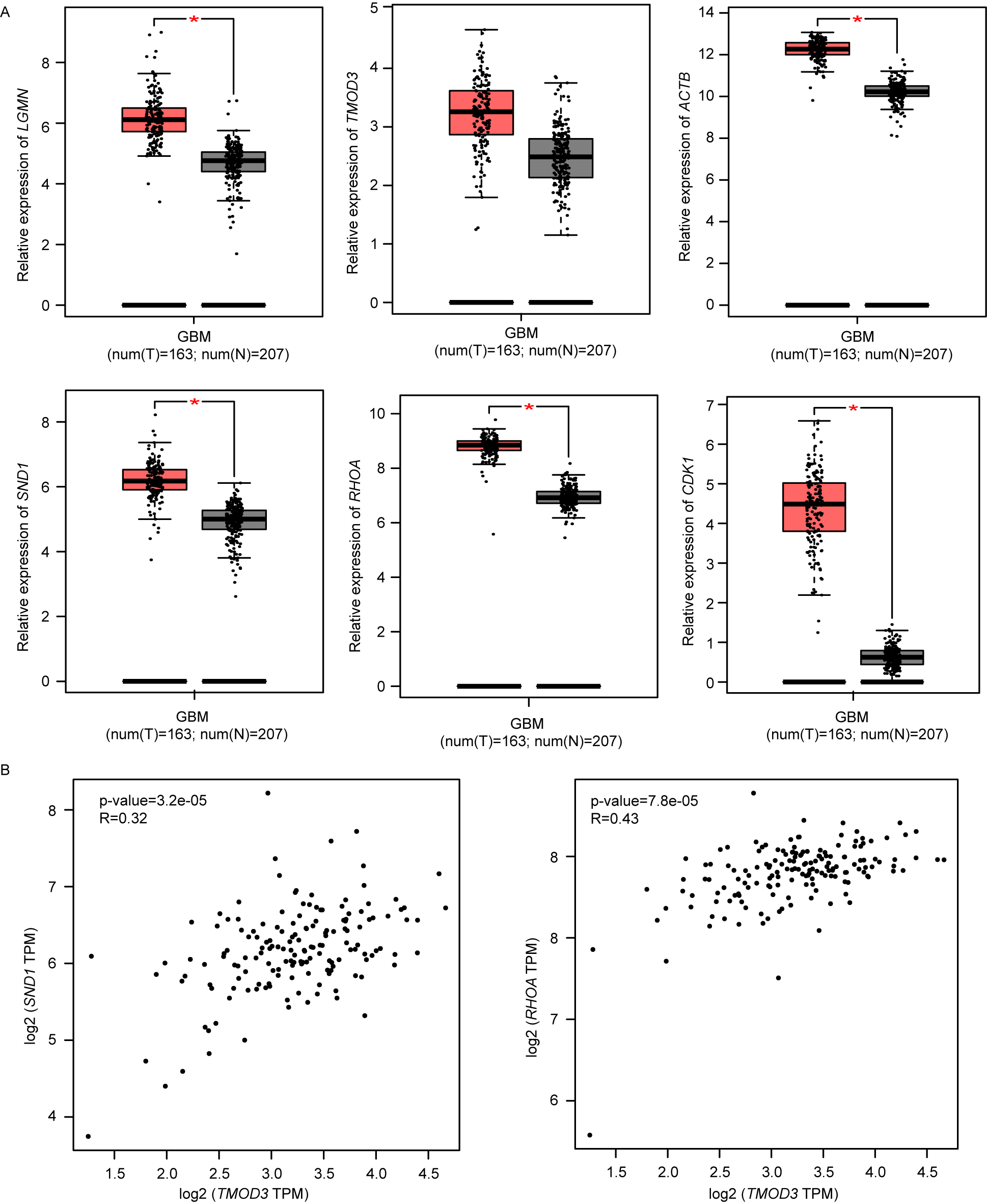

Supplement: Supplementary file 10 — Additional file 10: Fig. S10.Correlation analysis of Tmod3 and SND1/RhoA signaling. (A) The relative expression of LGMN, TMOD3, ACTB, SND1, RHOA and CDK1 in GBM compared to normal brain tissues by GEPIA analysis. (B) Correlation analysis of TMOD3 and SND1 or TMOD3 and RHOA expression in GBM by GEPIA analysis.*P<0.05. [file 13046_2022_2411_MOESM10_ESM.tif]
