# Supplementary material for: Cleavage of tropomodulin-3 by asparagine endopeptidase promotes cancer malignancy by actin remodeling and SND1/RhoA signaling
Source: J Exp Clin Cancer Res. 2022 Jun 28;41:209. doi: 10.1186/s13046-022-02411-4 (PMC9238189; doi:10.1186/s13046-022-02411-4)
Supplement: Supplementary file 16 — Additional file 16. Supplementary materials and methods. [file 13046_2022_2411_MOESM16_ESM.docx]

**Key Resources Table**

| **Regent or Resources** | **Source** | **Identifier** |
| --- | --- | --- |
| **Antibodies** |  |  |
| Tmod3 | Proteintech | Cat#12521-1-AP; RRID: AB2205324 |
| AEP | R&D Systems, | Cat#AF2199; RRID: AB416565 |
| Flag | Sigma-Aldrich | Cat#F1804; RRID: AB262044 |
| LAMP2 | Abcam | Cat#ab25631; RRID: AB470709 |
| ZsGreen1 | BBI | Cat#D199984 |
| Ki-67 | Abcam | Cat#ab16667; RRID: AB302459 |
| Vinculin | Cell Signaling Technology | Cat#4650, RRID: AB10559207 |
| SND1 | Proteintech | Cat#60265-1-Ig; RRID: AB2881386 |
| GAPDH | Abcam | Cat#ab9484; RRID: AB307274 |
| β-actin | Abcam | Cat#ab8226; RRID: AB306371 |
| β-tubulin | Abcam | Cat#ab21058; RRID: AB727045 |
| P53 | Santa Cruz Biotechnology | Cat#sc-126; RRID: AB_628082 |
| RhoA | Proteintech | Cat#10749-1-AP; RRID: AB2285104 |
| CDK4 | Proteintech | Cat#11026-1-AP; RRID: AB2078702 |
| CDK2 | Proteintech | Cat#10122-1-AP; RRID: AB2078556 |
| CDK1 | Proteintech | Cat#19532-1-AP; RRID: AB10638617 |
| Cyclin D1 | Proteintech | Cat#60186-1-Ig; RRID: AB10793718 |
| **Biological Samples** |  |  |
| Human glioma tissues | This paper | N/A |
| Human cervical cancer tissues | This paper | N/A |
| Human [hepatocellular](javascript:;) carcinoma tissues | This paper | N/A |
| Human normal brain tissues | This paper | N/A |
| **Chemicals, Peptides, and Recombinant Proteins** |  |  |
| LysoTracker Red DND-99 | Yeasen Biotechnology (Shanghai) Co., Ltd. | Cat#40739ES50 |
| His-tagged Tmod3 | This paper | N/A |
| His-tagged tTmod3-C | This paper | N/A |
| His-tagged tTmod3-N | This paper | N/A |
| Actin Polymerization Biochem Kit | Cytoskeleton, Inc. | Cat#BK003 |
| YF633 Dye Phalloidin Conjugates | US EVERBRIGHT INC | Cat#YP0053 |
| Deoxyribonuclease I | Invitrogen | Cat#D12372 |
| Nickel Magnetic Beads | Millipore | Cat#LSKMAGH02 |
| Nuclear and Cytoplasmic Protein Extraction Kit | Beyotime Biotechnology | Cat#P0027 |
| Leptomycin B | MedChemExpress | Cat#87081-35-4 |
| **Experimental models: Cell lines** |  |  |
| Human: HEK293 | ATCC | Cat# CRL-3216, RRID: CVCL_0063 |
| Human: U87-MG | ATCC | Cat# 300367/p658_U-87_MG, RRID: CVCL_0022 |
| Human: A172 | ATCC | Cat# CRL-7899; RRID: CVCL_0131 |
| Human: U251-MG | National Collection of Authenticated Cell Cultures | Cat#TCHu 58 |
| Human: HeLa | National Collection of Authenticated Cell Cultures | Cat#TCHu187 |
| **Software and Algorithms** |  |  |
| GraphPad Prism 7 | Graphpad Software | www.graphpad.com/scientificsoftware/prism/ |
| SPSS Statistics 14.0 | IBM | https://www.ibm.com/cn-zh/analytics/spss-statistics-software |
| Image J | N/A | https://imagej.nih.gov/ij/ |
| Image Pro Plus 7.0 | Media Cybernetics | N/A |
| Pannoramic Scanner | Pannoramic DESK, P-MIDI, 3D HISTECH, Hungary | N/A |
| Zeiss (LSM 510) software | Zeiss | N/A |
|  |  |  |

**Resource Availability**

Further information and requests for resources and reagents should be directed to and will be fulfilled by the lead contact, Yingying Lin ([yylin@sibs.ac.cn](mailto:yylin@sibs.ac.cn))

**Method Details**

**Coomassie Blue Staining and Silver Staining**

Protein lysis were separated by polyacrylamide gel electrophoresis (PAGE), the gels were put into an appropriate amount of Coomassie Blue staining solution (GelCode Blue reagent, Thermo Fisher Scientific) to ensure that the staining solution can fully cover the gel. After slowly shaking on a shaker, dye for 1 hour at room temperature, solution was replaced by the same volume of destaining solution, and the destaining solution should change several times until the blue background was completely washed. Final gels should be placed against a colorless background to capture images.

For silver staining, a Quick Silk Staining Kit (Beyotime Biotechnology, Shanghai, China) was used. Gels should be fixed in the stationary liquid for more than 20 minutes, then washed with 30 % ethyl alcohol, after that, wash again with Milli-Q grade pure water or double distilled water, sensibilization, washed with water twice, subsequently stained with silver solution (1 ×$x$) at room temperature for 10 min. Next, washed with water for 1 min, add silver dye solution, shake on a shaker at room temperature for 3-10 minutes until the desired protein band appears. Eventually, stop the reaction by stop solution (1 ×$x$), and images are captured.

**Single cell isolation and single cell/nuclear-seq library preparation**

Working on ice throughout. Single-cell/nuclear RNA sequencing was performed on a tumor biopsy specimen collected from patients with GBM or cervical cancer treated at the Ren Ji Hospital, Shanghai Jiao Tong University in Shanghai or Obstetrics and Gynecology Hospital of Fudan University School of Medicine. The biopsy specimens from the patient were banked for research purposes following consent through an IRB-approved protocol.

Libraries were prepared following the manufacturer’s protocol of Chromium Next GEM Single Cell 3ʹ Reagent Kits v3.1. Briefly, GEMs are generated by combining barcoded Single Cell 3ʹ v3.1 Gel Beads, a Master Mix containing cells, and Partitioning Oil onto Chromium Next GEM Chip G. To achieve single cell resolution, cells are delivered at a limiting dilution, such that the majority (~90-99 %) of generated GEMs contain no cell, while the remainder largely contain a single cell. Immediately following GEM generation, the Gel Bead is dissolved, primers are released, and any copartitioned cell is lysed. Mixed with the cell lysate and a Master Mix containing reverse transcription (RT) reagents Incubation of the GEMs produces barcoded, full-length cDNA from poly-adenylated mRNA. After incubation, GEMs are broken and pooled fractions are recovered. Silane magnetic beads are used to purify the first-strand cDNA from the post GEM-RT reaction mixture. Barcoded, full-length cDNA is amplified via PCR to generate sufficient mass for library construction. after cDNA Cleanup by SPRIselect Reagent and the resulting whole-transcriptome amplification measured by Qubit dsDNA Assay Kit (Life Technologies: Q328520) and quality assessed by BioAnalyzer (Agilent 2100). The Enzymatic fragmentation, End Repair & A-tailing and the following Post Ligation is following the manufacturer’s protocol. After the Sample Index PCR, the resulting libraries were quantified again by Qubit and BioAnalyzer. Libraries were pooled and sequenced.

**Single-cell/nuclear RNA-seq data preprocessing**

The Cell Ranger software pipeline (version 3.1.0) provided by 10×Genomics was used to demultiplex cellular barcodes, map reads to the genome and transcriptome using the STAR aligner, and down-sample reads as required to generate normalized aggregate data across samples, producing a matrix of gene counts versus cells. We processed the unique molecular identifier (UMI) count matrix using the R package Seurat (version 3.1.1). To remove low quality cells and likely multiplet captures, which is a major concern in microdroplet-based experiments, we applied criteria to filter out cells with UMI/gene numbers out of the limit of mean value +/- 2 folds of standard deviations assuming a Guassian distribution of each cells' UMI/gene numbers. Following visual inspection of the distribution of cells by the fraction of mitochondrial genes expressed, we further discarded low-quality cells where >10 % of the counts belonged to mitochondrial genes. After applying these QC criteria, 51538 single cells in GBM and 40000 single cells in cervical cancer were included in downstream analyses. Library size normalization was performed with Normalize Data function in Seurat to obtain the normalized count. Specifically, the global-scaling normalization method “LogNormalize” normalized the gene expression measurements for each cell by the total expression, multiplied by a scaling factor (10,000 by default), and the results were logtransformed. Top variable genes across single cells were identified using the method described in Macosko et al. The most variable genes were selected using FindVariableGenes function (mean. function = ExpMean, dispersion. function = LogVMR) in Seurat. Principal component analysis (PCA) was performed to reduce the dimensionality with RunPCA function in Seurat. Graph-based clustering was performed to cluster cells according to their gene expression profile using the FindClusters function in Seurat. Cells were visualized using a 2-dimensional t-distributed stochastic neighbor embedding (t-SNE) algorithm with the RunTSNE function in Seurat. The sequencing and bioinformatics analysis were performed by Oebiotech Co., Ltd. (Shanghai, China).

**Cell Counting Kit-8 (CCK-8) assay**

2×10^3^/100 μl cells were seeded on 96-well plates, three repeating wells for each group of experiments. 10 μl CCK-8 reagent was added into each well at 1-, 2-, 3-,4-, and 5-day. After 2 h incubation at 37 °C cell incubator, the 96-well plate was analyzed using a microplate reader (BioTek Flx800T, USA) at 450 nm.

**Flow cytometry for apoptosis detection**

Apoptosis was analyzed by translocation of phosphatidylserine to the cell surface using an Annexin and DAPI apoptosis detection kit (BD Biosciences, Franklin Lakes, NJ, USA). Cells were collected and washed with cold PBS. Then resuspended in Annexin V-FITC and DAPI for 30 min in the dark. Cell apoptosis was analyzed on a FACSAria flow cytometer (BD Biosciences) and quantified using CellQuest software 5.1 (BD Biosciences). Fluorescence was captured with an excitation wavelength of 480 nm.

**Colony Formation Assay**

1 ml of 0.8 % agar (low melting point agarose) was added to each well (6-well plate) and allowed to set as the base agar. Then, cells were trypsinized, and 500 cells/well (0.5 ml) were seeded. For plating, 0.5 mL pre-warmed DMEM with 20 % FBS and 0.5 ml 0.8 % agar was added to tubes with cells. Mix the mixture gently, then add 1ml to each experimental well of six-well plate. The assay was incubated at 37 ℃ in a humidified incubator for 10-14 days. Wells were stained with 1mL of 0.1 % crystal violet for 1 h before counting colonies under a dissecting microscope. The assays were repeated in triplicate

**Transwell Assay**

1×10^4^/100 μl cells were plated in the upper chambers of Matrigel-coated Transwell assay inserts (Millipore, Billerica, MA) in 200 μl serum-free DMEM medium (Hyclone, UT, USA). The assays were repeated in triplicate. The inserts were then placed into the bottom chamber of a 24-well plate containing DMEM medium with 10 % FBS as a chemoattractant. After 24 h, the top layer of the insert was scrubbed with a sterile cotton swab and the invading cells on the bottom surface were stained with 0.1 % crystal violet. The number of cells were examined, counted and imaged using digital microscopy. Five random fields of each chamber cells were counted, and an average number of cells were calculated.

**Scratch Wound-healing Assay**

Draw parallel horizontal lines on the back of the six-well plate, with at least 3 horizontal lines in each well, add the appropriate amount of uniformly mixed cells to the wells, the cells were 100 % confluent after overnight culture. Use a 200 μl pipette tip to draw in the well a straight line perpendicular to the parallel line on the back, washed with PBS twice to remove the sloughed cells, and then add serum-free medium to continue culturing. Take pictures at 24 h, 48 h or 72 h, and calculate the healing of the wounds.

**Hematoxylin-Eosin (H&E) and Immunohistochemical (IHC) Staining**

For H&E staining, paraffin embedded sections are dyed with hematoxylin for 5 minutes after they are deparaffinized and hydrated. Then washed with soft running water, treated with 1 % hydrochloric acid alcohol for seconds and quickly turn blue with 0.6 % ammonia. After that the slices are dyed in the eosin dye solution for 3min. After dehydration, use neutral resin to seal, and the images are captured by Pannoramic Scanner (Pannoramic DESK, P-MIDI, 3D HISTECH, Hungary). For IHC staining, paraffin-embedded sections were routinely deparaffinized and rehydrated similarly to the H&E staining. For antigen retrieval, the slides are heated at 98 ℃ in citrate buffer (pH 9.0) for 20 min and cooled to room temperature. After incubation with primary and secondary antibodies, the sections were stained using a highly sensitive streptavidin-biotin-peroxidase detection system and counterstained with hematoxylin. Primary antibodies used were listed as follow: AEP (R&D Systems, Cat. AF2199, dilution ratio 1:400), Tmod3 (Proteintech, Cat. 12521-1-AP, dilution ratio 1:400), Ki-67 (Abcam, ab16667, dilution ratio 1:400), Vinculin (Cell Signaling Technology, Cat. 4650, dilution ratio 1:400). For calculating integrated optic density (IOD) values of Tmod3 and Vinculin in tumor tissues, five representative fields (magnification$\times$200) in the region of tumors were randomly selected, Image‐Pro Plus 6.0 software (MEDIA Cybernetics, Rockville, MD, USA) was utilized. To analyze the proportion of Ki-67 positive cells in tumor tissues, five representative fields (magnification $\times$200) in the region of tumors were randomly selected, and cells with Ki-67 staining were counted.
